# Providing multimedia information to children and young people increases recruitment to trials: pre-planned meta-analysis of SWATs

**DOI:** 10.1186/s12916-023-02936-1

**Published:** 2023-07-04

**Authors:** Peter Knapp, Thirimon Moe-Byrne, Jacqueline Martin-Kerry, Rebecca Sheridan, Jenny Roche, Elizabeth Coleman, Peter Bower, Steven Higgins, Catherine Stones, Jonathan Graffy, Jenny Preston, Carrol Gamble, Bridget Young, Daniel Perry, Annegret Dahlmann-Noor, Mohamed Abbas, Payal Khandelwal, Siobhan Ludden, Augusto Azuara-Blanco, Emma McConnell, Nicky Mandall, Anna Lawson, Chris A. Rogers, Helena J. M. Smartt, Rachael Heys, Simon R. Stones, Danielle Horton Taylor, Sophie Ainsworth, Jenny Ainsworth

**Affiliations:** 1 Department of Health Sciences & the Hull York Medical School, University of York, York, UK; 2 Department of Health Sciences, University of York, York, UK; 3 York Trials Unit, Department of Health Sciences, University of York, York, UK; 4 Centre for Primary Care, University of Manchester, Manchester, UK; 5 School of Education, University of Durham, Durham, UK; 6 School of Design, University of Leeds, Leeds, UK; 7 Cambridge, UK; 8 Institute of Child Health, University of Liverpool, Liverpool, UK; 9 Department of Biostatistics, University of Liverpool, Liverpool, UK; 10 Department of Psychology, University of Liverpool, Liverpool, UK; 11 Nuffield Department of Orthopaedics, University of Oxford, Oxford, UK; 12 Moorfields Eye Hospital NHS Foundation Trust, London, UK; 13 Bedfordshire Community Health Services, Bedford, UK; 14 NIHR Moorfields Biomedical Research Centre, London, UK; 15 Queens University Belfast, Belfast, UK; 16 Tameside NHS Acute Foundation Trust, Manchester, UK; 17 Clinical Trials Unit, University of Birmingham, Birmingham, UK; 18 Trials Unit, University of Bristol, Bristol, UK; 19 Envision Pharma Group, Wilmslow, UK; 20 London, UK; 21 Liverpool, UK

**Keywords:** Children, Trial, Recruitment, Retention, Consent, Multimedia information

## Abstract

**Background:**

Randomised controlled trials are often beset by problems with poor recruitment and retention. Information to support decisions on trial participation is usually provided as printed participant information sheets (PIS), which are often long, technical, and unappealing. Multimedia information (MMI), including animations and videos, may be a valuable alternative or complement to a PIS. The Trials Engagement in Children and Adolescents (TRECA) study compared MMI to PIS to investigate the effects on participant recruitment, retention, and quality of decision-making.

**Methods:**

We undertook six SWATs (Study Within A Trial) within a series of host trials recruiting children and young people. Potential participants in the host trials were randomly allocated to receive MMI-only, PIS-only, or combined MMI + PIS. We recorded the rates of recruitment and retention (varying between 6 and 26 weeks post-randomisation) in each host trial. Potential participants approached about each host trial were asked to complete a nine-item Decision-Making Questionnaire (DMQ) to indicate their evaluation of the information and their reasons for participation/non-participation. Odds ratios were calculated and combined in a meta-analysis.

**Results:**

Data from 3/6 SWATs for which it was possible were combined in a meta-analysis (*n* = 1758). Potential participants allocated to MMI-only were more likely to be recruited to the host trial than those allocated to PIS-only (OR 1.54; 95% CI 1.05, 2.28; *p* = 0.03). Those allocated to combined MMI + PIS compared to PIS-only were no more likely to be recruited to the host trial (OR = 0.89; 95% CI 0.53, 1.50; *p* = 0.67). Providing MMI rather than PIS did not impact on DMQ scores. Once children and young people had been recruited to host trials, their trial retention rates did not differ according to intervention allocation.

**Conclusions:**

Providing MMI-only increased the trial recruitment rate compared to PIS-only but did not affect DMQ scores. Combined MMI + PIS instead of PIS had no effect on recruitment or retention. MMIs are a useful tool for trial recruitment in children and young people, and they could reduce trial recruitment periods.

**Supplementary Information:**

The online version contains supplementary material available at 10.1186/s12916-023-02936-1.

## Background

High-quality randomised controlled trials involving children and young people (CYP) are essential to ensure that interventions are safe and effective [1–4]. However, there is a lack of trial evidence, partly caused by poor recruitment, causing delayed completion and/or discontinuation [5, 6], and participant attrition [7, 8]. This contributes to trial costs [9] and research waste [10, 11]. Recruitment of CYP to trials is complicated by issues with consent and assent, judgements of ‘competence’, and different legal requirements for Clinical Trial of an Investigational Medicinal Product (CTIMP) and non-CTIMP trials [12, 13]. CYP may often be in a better position than their parent/carer to envisage what participation will mean [14, 15] and so excluding them from decision-making may lead to misunderstandings and subsequent withdrawal. Finally, when parents or carers are consenting on behalf of a child, the consent threshold tends to be higher [16], and parents or carers and healthcare professionals both tend to be more risk-averse (opting for standard care rather than a trial more often than they would for an adult) [17]. In 2013, the UK Chief Medical Officer [18] called for researchers to ‘…work with children and young people to input to the design of clinical studies…to facilitate (their) increased participation in trials’, an approach that has been undertaken in this study.

Information plays a crucial role in decision-making about trial participation [19]. In most cases, information is provided to potential participants as a printed participant information sheet (PIS); these have received prolonged criticism for being lengthy, technical, unappealing, and hard to navigate [20–23]. People with lower levels of literacy can find them especially difficult [24]. The UK Health Research Authority has encouraged researchers to use shorter PIS in low-risk research and explore the use of non-print media [25]. Providing multimedia information (MMI) through digital platforms could include animations, ‘talking-head’ videos, diagrams, photos, and written text [24, 26, 27].

In healthcare practice, MMIs have mostly been more effective than print for informing patients’ knowledge [28–31] such as about medical procedures [32–40]. The evidence in research recruitment is limited [26, 27], with only one study in CYP, which reported a greater understanding of trials from MMI [24]. In adults, the evidence is mixed: studies have reported increased understanding and knowledge of the trial [41] and positive participant evaluations [42], but another study reported that MMI and PIS produced similar trial recruitment rates [43].

MMIs have several potential advantages over PIS, including the following:

- Choice of the order in which aspects of the MMI are viewed
- Increased content choice, allowing some user personalisation
- Delivery by sound allows provision to those with sight impairment
- Concurrent delivery in sound and vision, facilitating user engagement and understanding, and reduced cognitive load [44]

**Fig. 1:**
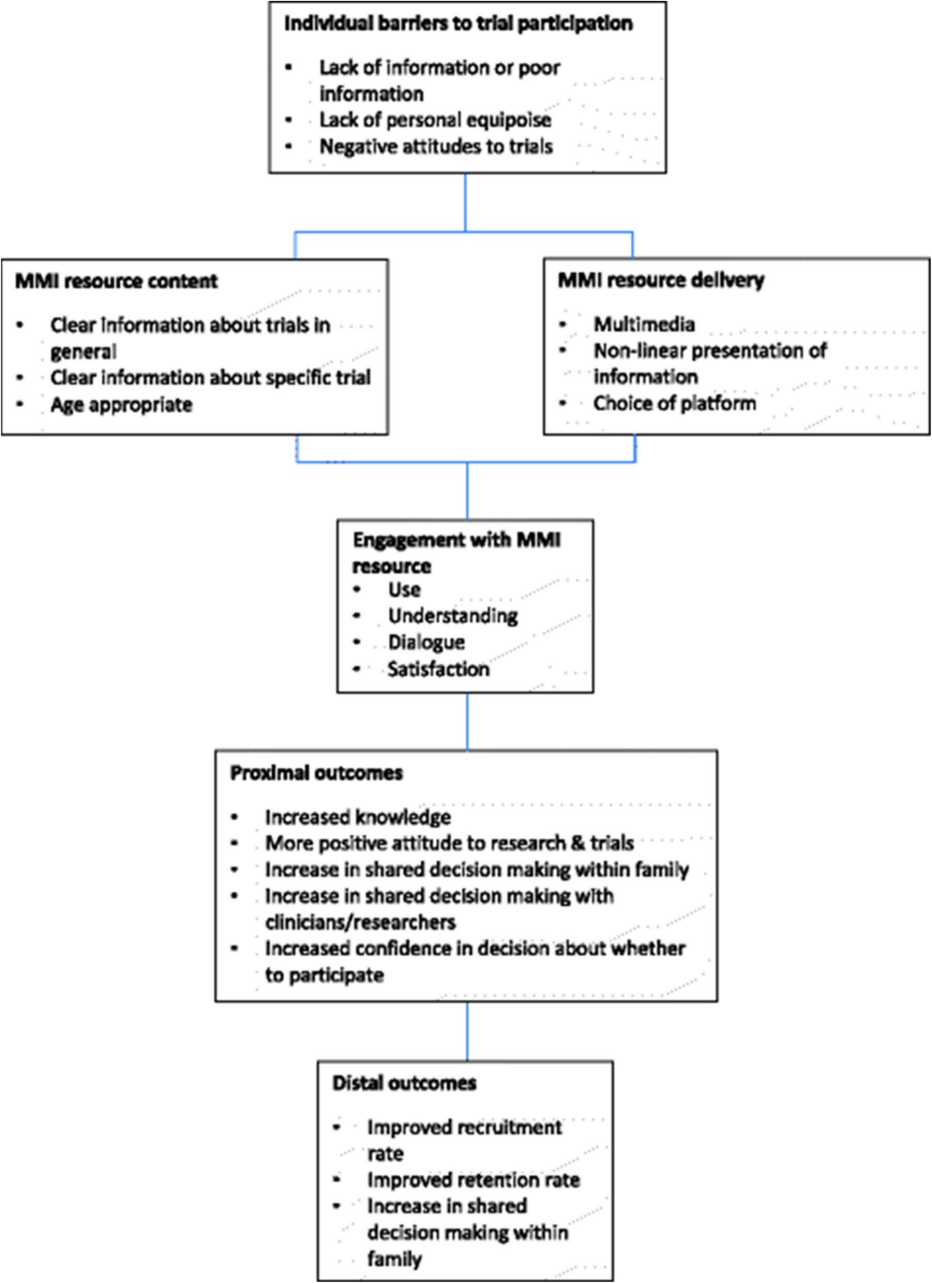
Logic model for the projected effects of multimedia information in trial recruitment

We suggest that positive effects of MMI on recruitment might be seen through more effective attention to, and use of the information, producing beneficial effects on knowledge, attitudes to trials, and decision confidence (Fig. 1).

The science of trial recruitment and retention is being developed through ‘Study Within A Trial’ (SWAT) methods, which are self-contained studies embedded within a host trial to evaluate alternative ways to deliver or organise trial processes [45–47]. The pre-planned coordination of SWATs is a recent development, first undertaken in MRC-START [48], and the approach has four main strengths:

1. Increased sample size and certainty of findings
2. Greater researcher control over participant sampling, outcomes, and (especially) interventions
3. Cost-efficiency, depending on the development time for the SWAT intervention(s)
4. Coordination of ‘recruitment science’, likely to accelerate evidence generation [45, 46]

The pre-planned approach to SWATs was undertaken in this study.

## Objectives

To evaluate MMI templates in a series of SWATs set within trials in CYP, to test their effects on recruitment and retention, and participant decision-making, by comparing the provision of MMIs to PIS, and the provision of MMIs in addition to PIS.

## Methods

**Fig. 2:**
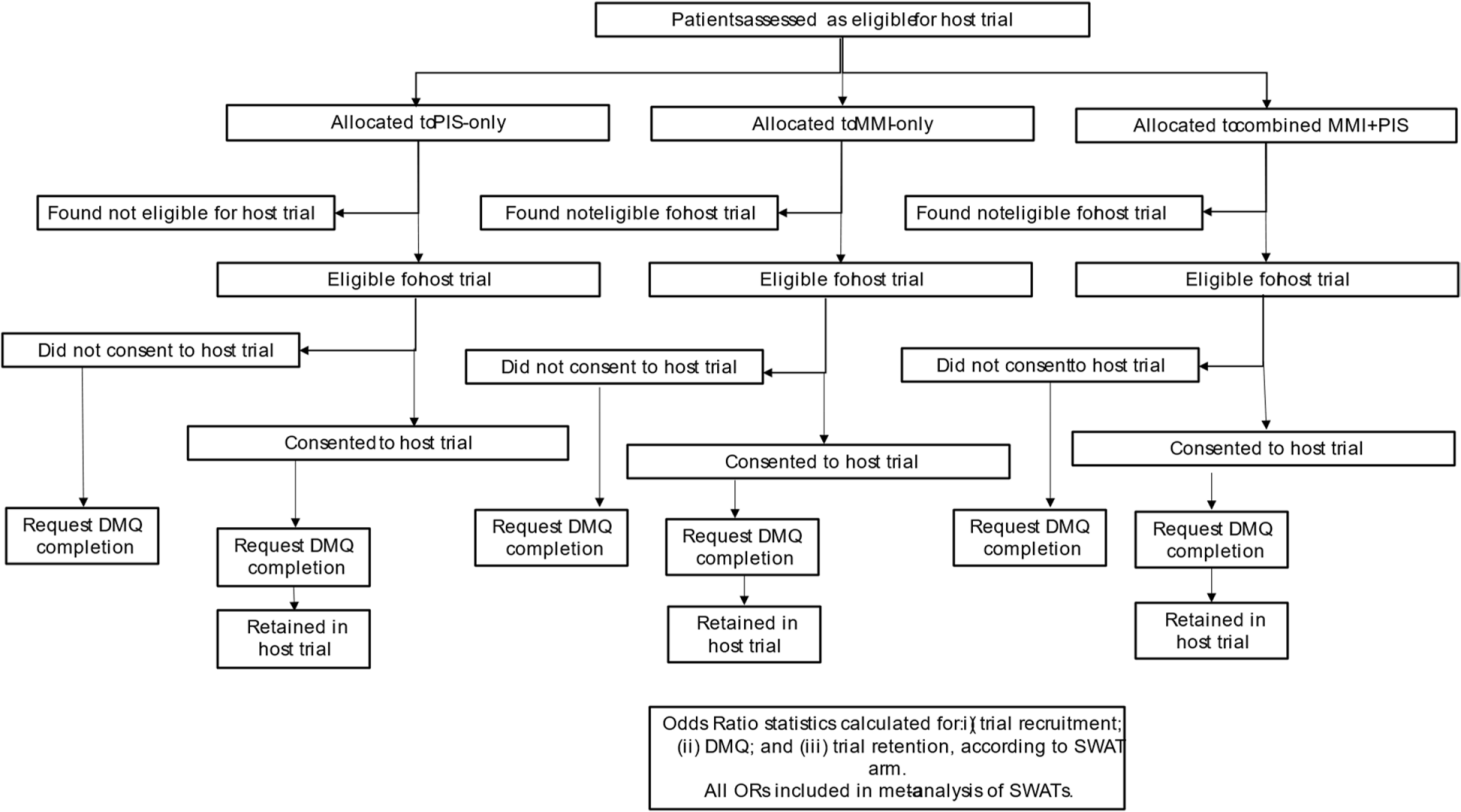
Pathway through each SWAT. *Note*: In the FORCE SWAT, only two allocations were used (MMI-only and PIS-only)

SWATs were embedded in a series of six host randomised control trials recruiting CYP. Within each SWAT, potential trial participants were randomised to receive trial recruitment information in one of three formats: MMI-only, PIS-only, or combined MMI + PIS (Fig. 2). Separate randomisation was done by each host trial, whereby their respective participants or recruiting sites (if cluster) were randomised to receive the information they had chosen to use in their trial. Details of the randomisation (ratios, clustering, stratification), and the arms included in each SWAT, can be found in Tables 1 and 2. Data from the SWATs were analysed and combined in a pre-planned two-stage meta-analysis.

**Table 1:** Summary of the six host trials

Trial	Target sample size (expected number to be approached, i.e. TRECA sample size)	Summary of host trial (funder; centres; eligible age range; health condition; interventions; stratification factors; retention time point)
FORCE	696 (1071)	NIHR HTA; multicentre; 4–16 years; Torus fracture; splint versus soft bandage immobilisation; age, centre; 6 weeks
CHAMP-UK	289 (413)	NIHR EME; multicentre; 6–12 years; myopia; atropine versus placebo; centre, ethnicity, severity of myopia; 6 months
THERMIC-3	94 (118)	British Heart Foundation; single-centre; 0–18 years; cardiac surgery; warm versus cold cardioplegia; risk adjustment for congenital heart surgery score; 3 months
BALANCE	66 (100)	Action for medical research; multicentre; 3–8 years; amblyopia; blurred movie watching versus standard patching/eye drops; type of amblyopia and centre; 16 weeks
BAMP	60 (100)	NHS Trust funding; single centre; 14–18 years; ‘reverse bite’; surgery at age 11–14 versus surgery at age 17 + ; gender; 18 months
UKALL-2011	40 (50)	Blood Cancer UK and Children with Cancer UK; multicentre; < 25 years; acute lymphoblastic leukaemia and lymphoblastic lymphoma; high-dose methotrexate versus standard maintenance therapy; maintenance with or without pulses R1: disease type (ALL/LBL), sex, age, white blood cell count R2: induction regimen, minimal residual disease risk group, disease type, sex, age, white blood cell count; 3 months

**Table 2:** Summary of the PIS and MMI used in each SWAT and the type of randomisation used

Trial	PIS and MMI summary
FORCE	**2 arms—cluster randomised, PIS or MMI (1:1)** **PIS:** 3 versions, one for parents/carers and two for CYP (younger and older). Developed with PPI representatives. The PIS for the parents/carers was 4 pages, and the CYP (younger and older) versions were 1 page and 2 pages, respectively **MMI:** 2 versions (6–11 years; adolescents and parents/carers). Contained all written content of the PIS, with text amended to improve clarity when required. Viewed on tablet computer at the hospital. Included 5 short video animations (each lasting 45–60 s) and 12 ‘talking head’ videos: 5 with a study investigator; 3 with a research nurse; plus 1 with an adolescent and 3 with parents/carers of CYP who had taken part in similar studies (each lasting 15–50 s) and describing different aspects of the trial. The MMI content was organised on 6 main webpages
CHAMP-UK	**3 arms—participant randomised, PIS, MMI, or PIS + MMI (1:1:1)** **PIS:** Emailed to participants prior to attending clinic for a screening/eligibility appointment. Three versions, one for parents/carers and 2 for children (including 1 that was a 2 pages picture booklet for CYP aged 6–7). Developed with PPI representatives. The PIS for parents/carers was 7 pages **MMI:** Link to URL to access MMI emailed to participants prior to attending clinic for a screening/eligibility appointment. Two versions (CYP; parents/carers), included 5 short video animations, each lasting 45–80 s. MMI contents for parents/carers organised on 6 main web pages: ‘Home page’, ‘About the study’, ‘Taking part’, ‘After the trial’, ‘Questions’, and ‘Contacts’. MMI for CYP had similar but less content under the 6 headings. The MMI for CYP included 19 ‘talking head’ videos: from the study investigator (13), CYP (5), and a parent from a similar study (1). Parents’/carers’ MMI included 19 ‘talking head’ clips (all < 1 min): from the study investigator (14), parents/carers (3), and CYP from a similar study (2)
THERMIC-3	**3 arms—participant randomised, PIS, MMI, or PIS + MMI (1:1:1)** **PIS:** Developed by the Thermic-3 trial team with feedback from the Generation R Young People’s Advisory Group (YPAG). Four versions, one for parents/carers and 3 for CYP (7–10, 11–15, 16–17 years). Length 2–7 pages **MMI:** Three versions (for different ages of CYP). The MMI was provided as a link (on laminated card), for viewing at the hospital or at home. Included 5 short video animations (each 45–60 s) and 34 ‘talking head’ video clips. The number of ‘talking head’ clips varied by age of intended user. The ‘talking head’ video clips (each lasting 10–80 s) were recorded with 4 individuals: a trial investigator, a research nurse, a parent/carer, and a CYP involved in a similar cardiac surgery study
BALANCE	**3 arms—participant randomised, PIS, MMI, or PIS + MMI (1:1:1)** **PIS:** Three versions (< 6 years, 6–8 years, parent/carers) and ranged from 1 to 9 pages according to age group **MMI:** Two versions (CYP; parents/carers). The MMIs included 5 short animation videos, each lasting 45–60 s. The parents’/carers’ MMI was organised on six main pages: ‘Home page’, ‘About the study’, ‘Taking part’, ‘After the trial’, ‘Questions’, and ‘Contacts’. The MMI for younger CYP had similar contents as the parents/carers. Both MMIs included 16 ‘talking head’ videos: study investigator (13), plus a young adult (1), and parent/carer (2) from a previous similar study (each lasting 20 s to 4 min)
BAMP	**3 arms—participant randomised, PIS, MMI, or PIS + MMI (1:1:1)** **PIS:** Two versions (parents/carers; CYP). Developed with PPI representatives. The PIS for parents was 7 pages, and the CYP’s PIS was 8 pages (one page for on assent form) **MMI:** Text addressed the CYP rather than the parent/carer (e.g. ‘your treatment’ rather than ‘their treatment’). The resource also included five short animation videos, each lasting 45–60 s (‘Summary of the key aspects of the BAMP trial’, ‘Why do we do trials?’, ‘What are trials?’, ‘Who’s in a trial team’, and ‘Assent and consent’) and 17 short ‘talking head’ videos, featuring 3 individuals (10 with the trial principal investigator; 4 with an adolescent who had received bone anchored maxillary protraction; 3 with a parent of a child who had received bone anchored maxillary protraction), each lasting 15–50 s and describing different aspects of the trial and clinical procedures. The MMI content was organised on 6 main webpages with the following headings: ‘Home page (including summary animation)’, ‘About the trial’, ‘Taking part’, ‘After the trial’, ‘Questions’, and ‘Contacts’
UKALL-2011	**3 arms—participant randomised, PIS, MMI, or PIS + MMI (1:1:1)** **PIS:** Five versions (under 8; 8–12 years; 13–15 years; 16 plus; parents/carers). Four to 9 pages according to age group **MMI:** The different versions of the MMIs were created for younger and older CYP/parents/carers and according to type of leukaemia or lymphoma. The MMIs also included 5 short animation videos, each lasting 45–80 s. The parents’/carers’ MMI contents were organised on 6 main web pages: ‘Home page’, ‘About the study’, ‘Taking part’, ‘After the trial’, ‘Questions’, and ‘Contacts’. The younger age group MMI had less content under each heading. The MMIs for younger patients included 17 ‘talking head’ videos: with the study investigator (16) and a parent of a child from a similar study (1). The MMIs for older patients and parents included 22 ‘talking head’ videos: from the study investigator (18) parents of a child from a similar study (4). Each clip lasted 20 s to 3 min

### Recruitment of host trials

We publicised the Trials Engagement in Children and Adolescents (TRECA) study through printed flyers and email to UK trial centres and the UK Trial Managers’ Research Network. We emphasised that the cost of developing the MMI would be borne by TRECA, and we also offered host trials modest financial support (£1500) to compensate for additional administrative work. Interested trials were selected based on the following:

- Recruitment timing (within the TRECA period)
- Not currently using MMI, website, or video in trial recruitment
- Recruiting CYP capable of at least some involvement in consent decisions (set as age 6 years and above)

Ideally, we wanted host trials to cover a range of health conditions and intervention types and be recruiting CYP across a range of ages. Given the historical prominence of oncology trials in CYP trial research [49], we preferred at least one host trial to have that setting.

Finally, the sample size calculation for the meta-analysis (see below) meant that host trials needed to approach an average of at least 329 CYP about participation. It is important to note that in these recruitment SWATs, the outcome of interest is the number of participants recruited out of the number of people approached to take part in the host trial. The people to be approached were randomly allocated to different recruitment methods, and the outcome is calculated according to the proportion of people recruited to the host trial from each arm in the SWAT.

Host trials were offered SWATs with two arms (MMI-only versus PIS-only; or MMI-only versus PIS + MMI) or three arms (MMI-only versus PIS-only versus PIS + MMI).

### Interventions

Interventions used in the TRECA study are listed in Table 2. The PIS was the Research Ethics Committee-approved information being used in each host trial; the PIS content for each trial was not modified. The decision on who read the PIS (i.e. parent, child, both together, both separately) was made by individual participants.

The MMI for each trial was based on a template developed through co-design and extensive empirical work during the first year of the TRECA study, comprising a qualitative study with stakeholder groups [50]; a user testing study with CYP, parents, and carers [51]; and close collaboration with the TRECA Patient and Parent Involvement group (PPI) [52]. Two templates were developed (one for children aged 6–11 years and a second for children aged 12–18 years and parents). The MMIs were developed by a website and video production company (Morph Studios Ltd.) and contained all the content from the PIS, organised into six sections within the MMI (home page, about the trial, taking part, after the trial, questions, contacts) plus five short video animations with voiceover (one that was trial-specific, summarising the trial; four that were trial-generic, explaining different aspects of trials), and a series of short ‘talking heads’ videos (e.g. the trial principal investigator or a young participant talking about aspects of the trial including the experience of participation). None of the video clips included subtitles. The number of ‘talking heads’ videos varied among the SWATs (see Table 3 for links to example MMIs). The written text of each MMI was amended for clarity through readability indices [53]. The decision on who viewed the MMI (i.e. parent, child, both together, both separately) was made by individual participants.

**Table 3:** Links to MMIs tested in the SWATs

Trial	Summary (URL for MMI link)
FORCE	https://www.york.ac.uk/healthsciences/research/health-policy/research/force-summary/
CHAMP UK	https://www.york.ac.uk/healthsciences/research/health-policy/research/champ/
THERMIC-3	https://www.york.ac.uk/healthsciences/research/health-policy/research/thermic-summary/
BALANCE	https://www.york.ac.uk/healthsciences/research/health-policy/research/balance/
BAMP	https://www.york.ac.uk/healthsciences/research/health-policy/research/decision-making-projects/trials-engagement-in-children/bampstudy/
UKALL	https://www.york.ac.uk/healthsciences/research/health-policy/research/ukall/

### Outcome measures

The primary outcome was trial recruitment in the MMI-only versus the PIS-only arms, to test the effect of replacing printed information with MMI. Trial recruitment was assessed by the number of eligible participants who were recruited to the host trial, according to the allocated SWAT arm.

The secondary outcomes were as follows:

- Trial recruitment in the MMI-only versus the combined PIS + MMI arms, to test the effect of providing MMI in addition to PIS
- Trial retention in the MMI-only versus the PIS-only arms
- Trial retention in the MMI-only versus the combined PIS + MMI arms
  - Trial retention was assessed by the number of participants in the host trial who were retained in the trial at follow-up. When a trial had multiple primary outcome time points, we selected one to maximise similarity across the host trials.
- Decision-Making Questionnaire (DMQ) scores in the MMI-only versus the PIS-only arms
- DMQ scores in the MMI-only versus the combined PIS + MMI arms
  - Decision-making (i.e. evaluation of the information plus reasons for participation/non-participation) was assessed by the mean score on the Decision-Making Questionnaire (DMQ) scale (see Supplementary Materials 4 for the three versions of the DMQ). The DMQ measured the quality of decision-making by potential participants and was developed for the study because currently available measures were intended for adults or measured decisions about treatments. When a parent/carer was involved in the decision, we also asked them to complete the scale separately. The scale was adapted to facilitate completion by younger CYP. We aimed to obtain DMQ scores both from individuals who decided to participate in the host trial and those who declined. In those who decided to take part, the CYP and/or parent/carer were asked to complete the DMQ once the host trial participation documentation was completed, or it was emailed to them. In CYP who declined participation, they were asked to complete the DMQ in the clinic or it was posted or emailed to home, as appropriate.
  - The DMQ versions for older CYP (intended for ages 12 and over) and parents or carers contained the same number of questions, with slight changes in question phrasing. It contained nine questions (with five fixed-response options), and there were a further three free-text questions. The younger CYP version of the DMQ (intended for ages 6–11) was of a similar format, comprising three questions with fixed responses and three ‘free-text’ questions.
  - Answers to fixed-response questions were allocated values of 0–4. The values for each question were summed to create an overall score out of 36 (or out of 12 for the younger CYP version), in which higher scores represented more positive evaluations. Up to three missing responses were allowed on the 9-question scale, and one was allowed on the three-question version. A total score was calculated by replacing missing values with the mean score from the completed responses given by the participant. Any questionnaires with more than three (older CYP/parent/carer version) or one (younger CYP version) missing values, were not scored.

### Trial and SWAT registration

The TRECA study was registered on the ISRCTN registry (ISRCTN73136092) and the Northern Ireland Hub for Trials Methodology Research SWAT Repository (SWAT 97). The SWATs were undertaken through amendments to Research Ethics Committee approvals obtained by the host trial research teams.

### Data analysis

#### Sample size calculation

The TRECA sample size was based on the meta-analysis of the recruitment data from each SWAT on the primary outcome (MMI-only versus PIS-only). We assumed 80% power at 5% type I error (alpha rate).

Assuming the baseline recruitment rate (in the PIS-only arm) was 80%, to detect an increase to 88% in the MMI arm in a single randomised controlled trial (RCT) with 1:1 randomisation between arms, *n* = 329 per group was needed. We multiplied this number by three to account for the 3-arm randomisation in the SWATs (*n* = 987). We assumed the heterogeneity in observed effect across the trials (*I* ^2^ statistic) would be 50%, having the effect of doubling the sample size, deriving an overall sample of 1974 across the six SWATs. This calculation has been updated from that included in the initial published protocol, due to an inability to reproduce the calculation. However, this does not impact on the validity of the results as the sample size of the overall project was driven by the individual SWATs that were undertaken, which could not be pre-determined, and which had their own recruitment targets.

#### Analysis

All analyses were conducted in STATA v16, following the principles of intention-to-treat (ITT) with participants’ outcomes analysed according to their original, randomised group. A modified ITT (mITT) approach was used, whereby any participants who were subsequently found to be ineligible for the host trial, were not included in the analysis. Where it was possible for a participant to receive a SWAT allocation different to what they were randomised to (for example, randomised to MMI, but received PIS), we also undertook a per-protocol analysis; these results are not presented and can be found in the main publication [54]. The analysis, outcomes, and significance levels were pre-specified in a statistical analysis plan (see Supplementary Material 1) before the analysis was conducted. Model assumptions were checked.

As there was a single primary outcome, we did not need to adjust for multiplicity issues arising from the PIS arm being in all comparisons.

#### Baseline data

All participant baseline data were summarised descriptively by the SWAT arm, and no formal statistical comparisons were undertaken. Continuous measures were reported as means and standard deviations (after normality was checked), and categorical data were reported as counts and percentages. Baseline data for the host trials varied due to different trial data collection; all data that were collected have been reported. Baseline data were only available for participants who were randomised into each host trial (see Supplementary Material 2).

### Primary analysis

#### Recruitment: MMI-only versus PIS-only

Recruitment rates were compared using logistic regression, undertaken separately for each SWAT, with SWAT allocation (MMI or PIS) included as a covariate. The results from the regression have been presented as odds ratios, with associated 95% confidence intervals and *p*-values.

### Secondary analyses

#### Recruitment: MMI + PIS versus PIS-only

This analysis was undertaken in the same way as the primary analysis, including only those trials which included a combined MMI + PIS arm.

#### Retention

Retention was measured as the proportion of completed follow-ups, i.e. the number of participants retained at the first follow-up measurement of the host trial primary outcome variable, divided by the number who were due a follow-up. The retention rate was compared using logistic regression, with SWAT allocation and host trial allocation (for FORCE and CHAMP-UK only) included as covariates. When a host trial used stratification variables (Table 1) in the randomisation, these were included as covariates wherever possible. As for the recruitment analyses, two pairwise comparisons were used: MMI-only versus PIS-only, and MMI + PIS versus PIS-only. The results have been presented as odds ratios, with associated 95% confidence intervals, and *p*-values.

#### Quality of decision-making questionnaires

The responses to each question (including the number of missing responses) and the calculated total scores of the DMQ were summarised descriptively overall and presented by host trial, SWAT allocation and type of questionnaire (younger CYP, older CYP, or parent/carer).

As CYP and their parent/carer may have both completed a questionnaire, data from all three questionnaires were not combined, due to the lack of independence. Hence, scores for CYP (younger or older) and parents/carer questionnaires were analysed separately using linear regression, with SWAT allocation and host trial status (whether the participant was recruited) included as covariates. The mean difference has been presented with 95% confidence intervals.

The DMQ results were compared using a regression model for each SWAT, adjusted for SWAT allocation, and whether CYP consented to participate in the host trial. To assess the robustness of the method used to replace the missing values, a sensitivity analysis was conducted, where the analysis was repeated using only the questionnaires in which all nine questions were answered.

For all the above analyses, if the SWAT was cluster randomised, the analysis included cluster as a random effect.

### Meta-analyses

The results from each SWAT were combined in meta-analyses. A two-stage random-effects meta-analysis was used in each case, where the results from each model were combined using an inverse-variance approach; no further adjustments were made. For each of the outcomes, any available data were combined using a meta-analysis.

## Results

### Host trials

Six host trials were recruited to TRECA: FORCE, CHAMP-UK, Thermic-3, BALANCE, BAMP, and UKALL-2011 (Tables 1 and 2), recruiting CYP with a variety of ages and health conditions in the UK (Table 1). Three of the SWATs generated sufficient data for logistic regression models, which were combined in a pre-planned statistical meta-analysis (FORCE, CHAMP-UK, Thermic-3). Unfortunately, three SWATs were significantly affected by adverse circumstances: the BAMP trial closed early due to funding restrictions; the UKALL-2011 SWAT took many months to approve, which limited recruitment before the host trial closed; and recruitment to the BALANCE trial was closed for several months due to the COVID-19 pandemic. Consequently, it was not possible to run logistic regression models for these three SWATs, due to being too small (2 trials) or having insufficient variation in outcomes (1 trial). Descriptive results are provided for each of the trials. However, the meta-analysis was based on the three SWATs where models could be fitted (total *n* = 1758).

**Fig. 3:**
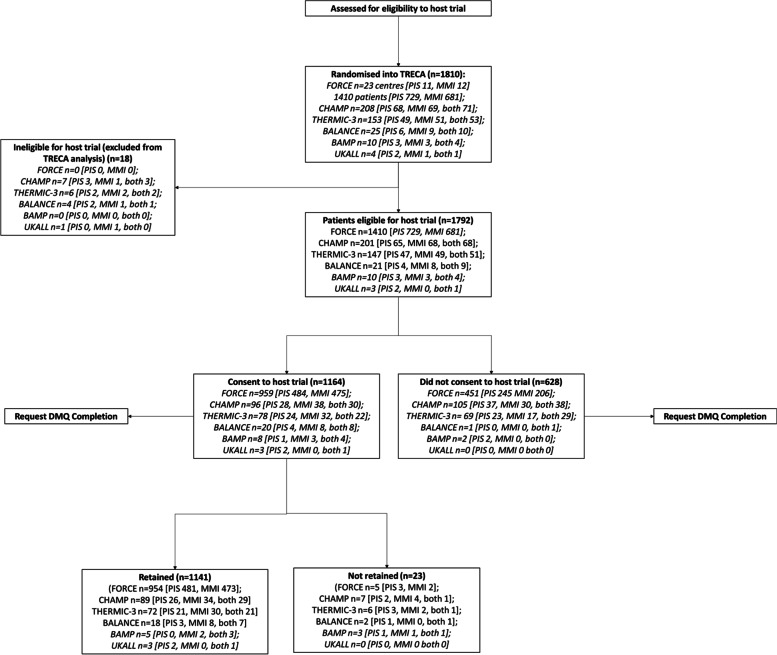
Flow of participants through each SWAT

The pathway through each SWAT is displayed in Fig. 3 and the results of the six individual SWATs are reported in Tables 4, 5, and 6. The results of the FORCE and Thermic-3 SWATs have already been reported individually [55, 56], and the results of the CHAMP-UK SWAT are reported in Supplementary Material 3.

**Table 4:** Summary of the recruitment analysis across the six SWATs, including the modified ITT meta-analysis results

Trial	PIS-only, *n*/*n* (%)	MMI-only, *n*/*n* (%)	MMI + PIS, *n*/*n* (%)	Overall, *n*/*n* (%)	MMI-only vs PIS-only^a^	MMI + PIS vs PIS-only^a^
**FORCE**	484/729 (66.4%)	475/681 (69.8%)	–	959/1410 (68.0%)	OR: 1.35 (95% CI 0.76, 2.40), *p* = 0.30	–
**CHAMP-UK**	28/65 (43.1%)	38/68 (55.9%)	30/68 (44.1%)	96/201 (47.8%)	OR: 1.67 (95% CI 0.84, 3.32), *p* = 0.14	OR: 1.04 (95% CI 0.53, 2.07), *p* = 0.90
**THERMIC-3**	24/47 (51.1%)	32/49 (65.3%)	22/51 (43.1%)	77/139 (55.4%)	OR: 1.80 (95% CI 0.79, 4.10), *p* = 0.16	OR: 0.73 (95% CI 0.33, 1.61), *p* = 0.43
**BALANCE**	4/4 (100.0%)	8/8 (100.0%)	8/9 (88.9%)	20/21 (95.2%)	–	–
**BAMP**	1/3 (33.3%)	3/3 (100.0%)	4/4 (100.0%)	8/10 (80.0%)	–	–
**UKALL-2011**	2/2 (100.0%)	–	1/1 (100.0%)	3/3 (100.0%)	–	–
**Overall**	**543/850 (63.9%)**	**556/809 (68.7%)**	**65/133 (48.9%)**	**1164/1792 (65.0%)**	–	–
**Meta-analysis results**					**OR: 1.54 (95% CI 1.05, 2.28), ** ***p*** = 0.03**	**OR: 0.89 (95% CI 0.53, 1.50), ** ***p*** = 0.67***

^**^FORCE, CHAMP-UK, and THERMIC-3 are included

^***^CHAMP-UK and THERMIC-3 are included

^a^PIS-only is the reference class

**Table 5:** Summary of the retention analysis across the six SWATs, including the meta-analysis results

Trial	PIS-only, *n*/*n* (%)	MMI-only, *n*/*n* (%)	MMI + PIS, *n*/*n* (%)	Overall, *n*/*n* (%)	MMI-only vs PIS-only^a^	MMI + PIS vs PIS-only^a^
**FORCE**	481/484 (99.4%)	473/475 (99.6%)	–	954/959 (99.5%)	OR: 1.14 (95% CI 0.11, 12.32), *p* = 0.91	–
**CHAMP-UK**	26/28 (92.9%)	34/38 (89.5%)	29/30 (96.7%)	89/96 (93.0%)	OR: 1.11 (95% CI 0.12, 10.27), *p* = 0.92	OR: 2.23 (95% CI 0.19, 26.06), *p* = 0.52
**THERMIC-3**	21/24 (87.5%)	30/32 (93.8%)	21/22 (95.5%)	72/77 (94.0%)	OR: 1.62 (95% CI 0.20, 12.98), *p* = 0.65	OR: 2.05 (95% CI 0.17, 24.64), *p* = 0.57
**BALANCE**	3/4 (75.0%)	8/8 (100.0%)	7/8 (87.5%)	18/20 (90.0%)	–	–
**BAMP**	0/1 (0%)	2/3 (66.7%)	3/4 (75.0%)	5/8 (63.0%)	–	–
**UKALL-2011**	2/2 (100.0%)	–	1/1 (100.0%)	3/3 (100.0%)	–	–
**Overall**	**533/543 (98.2%)**	**547/556 (98.4%)**	**61/65 (93.8%)**	**1141/1164 (98.0%)**	–	–
**Meta-analysis results**					**OR: 1.29 (95% CI 0.36, 4.65), ** ***p*** = 0.70**	**OR: 2.18 (95% CI 0.48, 10.00), ** ***p*** = 0.31***

^**^FORCE, CHAMP-UK, and THERMIC-3 are included

^***^CHAMP-UK and THERMIC-3 are included

^a^PIS-only is the reference class

**Table 6:** DMQ mean and standard deviation for each trial, by TRECA allocation and DMQ version, and results of comparisons

Trial (DMQ versions used)	PIS-only	MMI-only	MMI + PIS	Overall	MMI-only vs PIS-only^a^	MMI + PIS vs PIS-only^a^
**FORCE (P/C)**	P/C: *N* = 157 31.2 (4.9)	P/C: *N* = 151 31.3 (4.5)	–	P/C: *N* = 308 31.3 (4.7)	P/C: *N* = 308 AMD: 0.07 (95% CI − 1.08, 1.22) *P* = 0.91	–
**CHAMP-UK (young and P/C)**	Young: – P/C: *N* = 26 33.2 (3.4)	Young: – P/C: *N* = 31 29.7 (4.6)	Young: – P/C: *N* = 24 30.0 (3.8)	Young: *N* = 1 P/C: *N* = 81 30.6 (4.1)	Young: – P/C: *N* = 57 AMD: − 2.43 (95% CI − 4.61, − 0.24) *P* = 0.03	Young: – P/C: *N* = 50 AMD: − 2.11 (95% CI − 4.23, 0.01) *P* = 0.05
**THERMIC-3 (all three)**	Young: – Older: – P/C: *N* = 8 29.5 (6.9)	Young: – Older: – P/C: *N* = 6 31.8 (4.4)	Young: – Older: – P/C: *N* = 3 28.3 (4.9)	Young: – Older: – P/C: *N* = 17 30.1 (5.7)	Young: – Older: – P/C: *N* = 14 AMD: 0.73 (95% CI − 5.34, 6.80)	Young: – Older: – P/C: *N* = 11 AMD: 0.72 (95% CI − 10.43, 7.53)
**BALANCE (young and P/C)**	Young: – P/C: –	Young: – P/C: –	Young: – P/C: –	Young: *N* = 4 8.8 (2.2) P/C: *N* = 9 30.1 (4.0)	Young: – P/C: –	Young: – P/C: –
**BAMP (older)**	Older: *N* = 3 29.0 (8.9)	Older: *N* = 3 33.7 (2.1)	Older: *N* = 4 31.3 (4.4)	Older: *N* = 10 31.3 (5.4)	Older: *N* = 6 AMD: 1.67 (95% CI − 24.59, 27.92) *P* = 0.85	Older: *N* = 7 AMD: − 0.75 (95% CI − 22.88, 21.37) *P* = 0.93
**UKALL-2011 (all three)**	Young: – Older: – P/C: –	Young: – Older: – P/C: –	Young: – Older: – P/C: –	Young: – Older: *N* = 2 19.5 (2.1) P/C: *N* = 3 21.3 (14.6)	Young: – Older: – P/C: –	Young: – Older: – P/C: –
**Overall**	**Young:** – **Older:** ***N*** **= 4** **26.3 (9.1)** **P/F:** ***N*** **= 217** **31.0 (4.9)**	**Young:** – **Older:** ***N*** **= 3** **33.7 (2.1)** **P/F:** ***N*** **= 166** **31.0 (5.6)**	**Young:** – **Older** **:** ***N*** **= 5** **29.2 (6.0)** **P/F:** ***N*** **= 28** **30.0 (4.0)**	***Young:*** ***N*** **= 5** **9.4 (2.4)** ***Older:*** ***N*** **= 12** **29.3 (6.7)** **P/F:** ***N*** **= 411** **30.9 (5.1)**		
**Meta-analysis results**					***Young:*** – ***Older:*** – ***P/C*** **AMD: − 0.79 (95% CI − 2.80, 1.22)** ***P*** = 0.44**	***Young:*** – ***Older:*** – ***P/C*** **AMD: − 2.07 (95% CI − 4.13, − 0.01)** ***P*** = 0.05***

^a^PIS-only is the reference class

^**^FORCE, CHAMP-UK, and THERMIC-3 are included

^***^CHAMP-UK and THERMIC-3 are included

### Meta-analysis of SWAT data

#### Trial recruitment

**Fig. 4:**
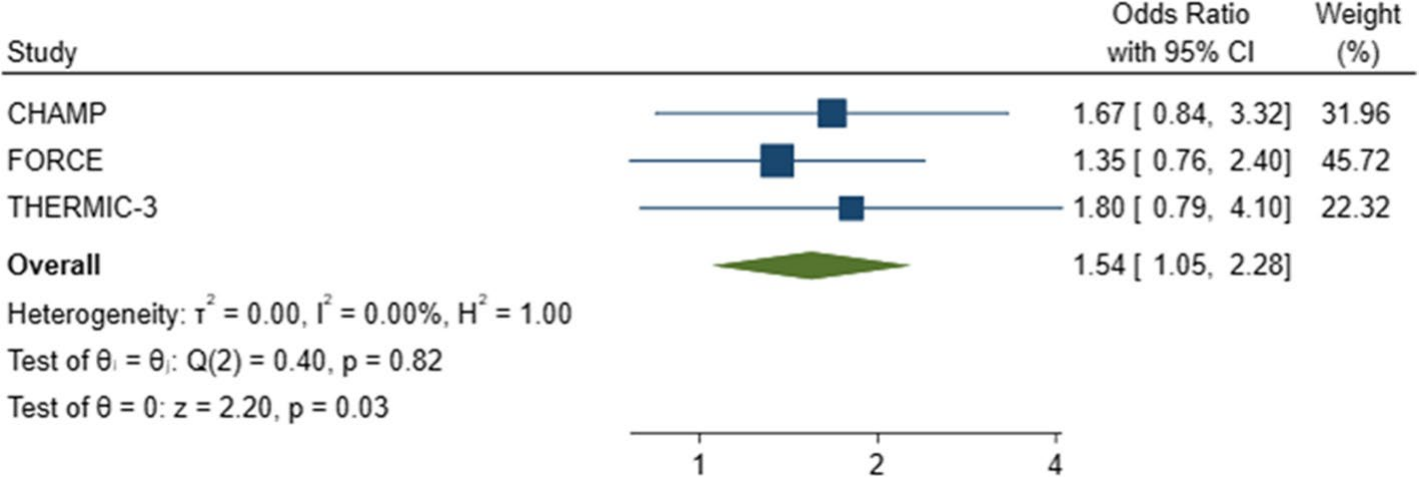
Forest plot of trial recruitment in the MMI-only and PIS-only arms for the mITT

The pooled results of the mITT data show that those CYP who received MMI-only information were more likely to be recruited into a trial than those who received PIS-only information: pooled odds ratio (OR) = 1.54 (95% CI: 1.05, 2.28; *p* = 0.03). The width of the 95% confidence interval indicates considerable uncertainty about the true effect. The statistical heterogeneity (*I* ^2^) in the meta-analysis was 0% (Fig. 4).

The meta-analysis comparing PIS-only versus the combined MMI + PIS arms included data from only two SWATs (CHAMP-UK and Thermic-3) because the FORCE SWAT did not include a combined MMI + PIS arm. There was no significant difference between the two arms: pooled OR = 0.89 (95% CI: 0.53, 1.50; *p* = 0.67).

#### Trial retention rates

Retention was recorded between 6 and 26 weeks post-randomisation, depending on the trial. The retention rates of CYP recruited to host trials in the MMI-only and PIS-only arms were similar: pooled OR = 1.29 (95% CI: 0.36, 4.65; *p* = 0.70). Statistical heterogeneity was 0% (Table 5).

The retention rate was higher in the MMI + PIS arm than in the PIS-only arm, but sample sizes were small and the difference was not statistically significant: pooled OR = 2.18 (95% CI: 0.48, 10.00; *p* = 0.31). Statistical heterogeneity was 0%.

#### Quality of decision-making questionnaires

The meta-analysis of the DMQs is limited to the parent/carer (P/C) version of the questionnaire because there were insufficient data for meta-analyses of the younger and older CYP versions. The results of the younger and older CYP versions are given descriptively where possible (see Table 6).

Overall, for the parent/carer version those in the MMI-only arm had similar (but slightly lower) DMQ scores to those in the PIS-only arm: pooled adjusted mean difference (AMD) =  − 0.79(95% CI: − 2.80, 1.22; *p* = 0.44). Statistical heterogeneity was moderately high (53.6%).

Participants in the combined MMI + PIS arm had lower DMQ scores than those in the PIS-only arm: pooled AMD =  − 2.07 (95% CI: − 4.13, 0.01; *p* = 0.05), which is of borderline statistical significance. Statistical heterogeneity was 0%.

## Discussion

### Brief summary of findings

MMI provision, rather than standard PIS, led to higher recruitment rates of CYP to trials. There was no effect on trial retention. Providing combined MMI + PIS resulted in no benefit on trial recruitment or retention rates compared to PIS alone.

The provision of MMI rather than PIS produced no effect on DMQ scores. However, the provision of combined MMI + PIS resulted in less favourable evaluations than from PIS alone.

### Strengths and limitations

The MMIs were developed through extensive empirical work, to ensure that their content, layout, and appearance would best meet the preferences and needs of CYP, parents/carers, healthcare practitioners, and researchers. Two templates were developed (one for children aged 6–11 and one for children aged 12–18 and parents), based on estimated literacy and cognitive maturity. Finer-grained age distinctions would be possible in MMIs although there would be both financial and practical implications.

The six host trials recruited CYP with a range of ages and health conditions across the UK, although we found that many otherwise eligible host trials were too small to include in TRECA. Unfortunately, we were able to include data from only three of the SWATs in the meta-analysis. However, the pre-planned meta-analysis increased the available sample size for evaluating the effects of MMIs, and allowed the comparison of similar interventions (i.e. template-driven MMIs) across several trials.

The study faced several challenges [57], the main effect of which was to reduce the size of the meta-analysis dataset. There was a good response to publicity about TRECA, but some potential host trials did not meet our criteria. In addition, trial recruitment postponements due to COVID-19 affected three SWATs, reducing the dataset size in two of them (by around *n* = 200 in total) and delaying the meta-analysis. Unfortunately, any adverse circumstance affecting host trial progress will almost always affect SWATs. On the other hand, the sample size estimate assumed a meta-analysis heterogeneity value of 50%, but in fact, a value of 0% was obtained, increasing power.

The DMQ return rates were around half that anticipated, reducing statistical precision. The questionnaire return rate was much higher in CYP recruited to host trials than in those who declined participation. It constitutes a significant limitation to the findings; potentially, it was also a source of bias in the DMQ dataset. Sending questionnaires via website or email (as happened in the CHAMP-UK trial), rather than providing printed versions, could have increased return rates, but this approach has not been evaluated [58]. We could also have sought other data sources on recruitment. For example, we could have interviewed people who did or did not consent in host trials, or clinical and research staff responsible for trial recruitment, although this approach would have had its own challenges.

Finally, a common limitation with SWATs is the application of host trial entry criteria to patient eligibility after the SWAT random allocation of individuals has happened. The effect is to lose SWAT participants and reduce the fidelity of the SWAT randomisation; consequently, a modified intention-to-treat (mITT) analysis was required in this study. In all, 18 participants were omitted from the analysis for being ineligible for the host trial (7 CHAMP-UK, 1 UKALL, 4 BALANCE, 6 THERMIC-3).

### What this study adds

This study contributes to the growing evidence base of RCT-level evidence for interventions targeting trial recruitment and retention [45], particularly in CYP, which is currently lacking.

The retention rates of CYP at follow-up in the trials are reassuring, indicating that increased recruitment through MMI was not achieved at the expense of understanding the host trial when being recruited.

One aim of the study was to produce an MMI template for trial recruitment, in part to ensure that all the TRECA SWATs would be testing similar multimedia information, and to allow future practitioners and researchers to use the template in trials if the MMI was shown to be effective and/or acceptable. That opportunity is now available, and access can be provided on request.

There is growing evidence on the use of MMI in research recruitment and healthcare, although few interventions have been evaluated within SWATs using random allocation. As such, this study makes a meaningful contribution.

Finally, this study shows that using MMI in research recruitment is achievable and acceptable. Our view is that a hesitancy among researchers towards digital or multimedia information stems from two concerns:

1. First, a view that Research Ethics Committees will be reluctant to approve non-print participant information. Notably, we did not encounter any such resistance during this study.
2. Second, that MMI is too difficult, expensive, or time-consuming to produce. Depending on its quality and complexity, a trial MMI currently costs £10,000–£15,000. The pooled absolute recruitment rate in this study was 4.8% higher in the MMI-only arm (68.7% versus 63.9%, see Table 4), which would equate to a relative reduction of 7% in the recruitment period in the host trials, potentially reducing study length and cost. If replicated, the use of MMIs in trials could be cost-neutral or even cost-saving. However, no formal health economic analysis was undertaken.

### Implications of the study

This study demonstrated a benefit of MMI on trial recruitment in CYP, although the relevant evidence base is small and further research is needed. Furthermore, the effect of MMIs on recruitment generated an odds ratio statistic with the lower end of the confidence interval not far above 1 (with an associated probability of 0.03) and so there remains considerable uncertainty around its true effect. That is particularly the case for CYP, but it also applies to trials recruiting adults.

The combined provision of MMI + PIS resulted in no gains, and possibly less positive questionnaire evaluations. However, the small available dataset for this question greatly lessens the certainty of this finding. Given the time demands and complexity of generating participant information in two formats, this study does not suggest that using both MMI and PIS is a better option, although it would be helpful for further research to assess this point.

One SWAT could not provide participants with MMI access as planned due to poor hospital internet connectivity [57]. Consequently, in that SWAT, participants were given the MMI URL on a laminated card, to access at home, increasing the risk of non-access. A solution could be through social media or email/text message communication between the healthcare provider and patients, although there are several barriers (ethical and practical) that may need to be overcome.

One aim was to assess the relative impacts of MMI and PIS on more deprived and/or less health-literate populations. Suboptimal or complex patient information tends to have a disproportionately negative effect on people with low literacy (including low health literacy) [24]. Unfortunately, we were unable to examine these aspects. Demographic baseline data are generally only available for those who have consented to the host trial. Due to research ethics restrictions, data access is rarely permitted for people who have declined trial participation, placing a significant limit on assessing equity within ‘recruitment science’. There remains a need to assess whether carefully developed MMI has similar benefits on understanding and decision-confidence across population groups. Easy internet access in the population is commonplace but not universal. There is a likelihood of deepening of inequities if information is exclusively provided via MMIs, or both MMIs and PIS are provided but MMI content has greater clarity. MMIs may also have negative associations for some users: video and animations can appeal and engage, although these effects must not be achieved through superficiality or imbalance. Content in different formats may lead to duplication or ‘information redundancy’ [59]. Finally, a recent review identified that CYP with long-term health conditions may associate digital health technologies with concerns about privacy, trust, and confidentiality [60].

Information plays an essential role in research consent. Valid participation decisions (whether positive or negative) must be informed, and the person taking consent has an ethical duty to be sure that the participant is making an informed decision. The role of MMI in individual consent decisions, and in allowing recruiters to fulfil their ethical duty, is an area in need of research. However, the external context is changing: people are increasingly expecting digital provision of information, particularly younger people, and digital provision may also be altering content expectations [61, 62]. These are two of the factors which may increase participant acceptance and use of MMI in trial recruitment.

This study has generated some relevant findings from the DMQs, although low return rates (particularly from those declining trial recruitment) mean that there is considerable uncertainty about MMI evaluations. For example, the findings of a SWAT undertaken within a hypothetical trial were that the TRECA-generated MMI produced higher appraisals of ‘information was easy to understand’ and ‘I had confidence in decision-making’ than did PIS provision [63]. There is also a need for research with adults and CYP around the effects of MMI on communication between potential participants and those recruiting them; MMIs may impact recruiter-participant interaction, but this needs evaluation.

We should also know more about people’s use of MMIs to inform decisions, to ensure they are being informed about research and not just being entertained. Furthermore, it is unclear whether all the components of the MMI are required to achieve benefits. Two concerns are that:

1. Animations and video are relatively expensive components of MMIs, which may create a barrier in less well-funded trials.
2. The inclusion in MMIs of all the written PIS text plus information content within the animations and videos allows choice but may create information duplication [59]. Analysis of the use of the various components of MMIs could indicate whether duplication and/or redundancy are apparent.

The overall effect of the MMIs in TRECA was to increase recruitment in children and young people (indicated by a meta-analysis of three SWATs with negligible statistical heterogeneity), which indicates their potential as a recruitment tool. However, given the width of the confidence intervals, and a dataset based on just three SWATs, there remains a need for more research to produce a more precise estimate of how well MMIs work and in what settings. The TRECA dataset on combined MMI + PIS was small and further research would increase the certainty on its effects. Finally, we need to explore what components of MMIs are associated with benefits, and whether MMIs are cost-effective.

## Conclusions

Providing only multimedia information increased the trial recruitment rate compared to providing only printed information, but it did not affect people’s evaluation of the information. Among children and young people recruited to host trials, trial retention rates did not differ according to intervention allocation. Providing combined multimedia and printed information instead of printed information only had no effect on recruitment or retention. Multimedia information is a useful tool for trial recruitment in children and young people, and it could reduce trial recruitment periods.

## Supplementary Information

**Additional file 1.** Statistical Analysis Plan.

**Additional file 2.** Baseline characteristics for participants who consented to the FORCE, CHAMP-UK and Thermic-3 trials.

**Additional file 3.** Results of the CHAMP-UK SWAT.

**Additional file 4.** Decision-making Questionnaire.

## Data Availability

Upon request, and subject to review, the authors will provide the data that support the findings of this study.

## Abbreviations

AMD: Adjusted mean difference
CI: Confidence interval
COVID-19: Coronavirus disease 2019
CYP: Children and young people
DMQ: Decision-Making Questionnaire
HCP: Healthcare professional
ITT: Intention-to-treat
mITT: Modified intention-to-treat
MMI: Multimedia information
OR: Odds ratio
PIS: Participant information sheet
RCT: Randomised controlled trial
SWAT: Study Within A Trial
TRECA: Trials Engagement in Children and Adolescents
UK: United Kingdom
USA: United States of America
