# Supplementary material for: Providing multimedia information to children and young people increases recruitment to trials: pre-planned meta-analysis of SWATs
Source: BMC Med. 2023 Jul 4;21:244. doi: 10.1186/s12916-023-02936-1 (PMC10320935; doi:10.1186/s12916-023-02936-1)
Supplement: Supplementary file 1 — Additional file 1. Statistical Analysis Plan. [file 12916_2023_2936_MOESM1_ESM.docx]

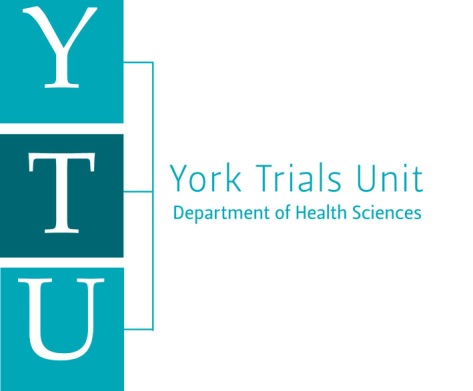


| **TRECA** |  |  |
| --- | --- | --- |
| **The TRECA study: TRials Engagement in Children and Adolescents** | | |
|  | | |
| STATISTICAL ANALYSIS PLAN | | |
| v1.4 | | |

**York Trials Unit Version date:** 21/01/2020
Department of Health Sciences **Author:** Jenny Roche & Caroline Fairhurst
University of York **Chief Investigator:** Dr Peter Knapp
York, YO10 5DD **Study Coordinator**: Thirimon Moe Byrne

Contents

[1. Definition of terms/acronyms 2](#_Toc26968298)

[2. Design 2](#_Toc26968299)

[2.1 TRECA 2](#_Toc26968300)

[2.2 Host Trials 2](#_Toc26968301)

[3. Trial Objectives 4](#_Toc26968302)

[4. Sample Size 4](#_Toc26968303)

[4.1 Overall 4](#_Toc26968304)

[4.2 BAMP hypothetical sub-study 5](#_Toc26968305)

[5. Randomisation 5](#_Toc26968306)

[6. Outcomes 5](#_Toc26968307)

[6.1 Primary outcome (Recruitment - MMI only vs PIS only) 5](#_Toc26968308)

[6.2 Secondary outcomes 5](#_Toc26968309)

[6.2.1 Recruitment (MMI & PIS vs PIS only) 5](#_Toc26968310)

[6.2.2 Retention 6](#_Toc26968311)

[6.2.3 Quality of Decision Making 6](#_Toc26968312)

[6.3 Other important information 7](#_Toc26968313)

[7. Analysis 7](#_Toc26968314)

[7.1 Baseline data 7](#_Toc26968315)

[7.2 Primary analysis (Recruitment - MMI only vs PIS only) 7](#_Toc26968316)

[7.3 Secondary analyses 8](#_Toc26968317)

[7.3.1 Recruitment (MMI & PIS vs PIS only) 8](#_Toc26968318)

[7.3.2 Retention 8](#_Toc26968319)

[7.3.3 Quality of Decision Making 8](#_Toc26968320)

[8. Meta-analyses 9](#_Toc26968321)

[8.1 Recruitment 9](#_Toc26968322)

[8.2 Retention 9](#_Toc26968323)

[8.3 Quality of decision making 9](#_Toc26968324)

[9. SAP amendment log 10](#_Toc26968325)

[10. Signatures of approval 10](#_Toc26968326)

[11. References 10](#_Toc26968327)

[12. Appendix 10](#_Toc26968328)

[12.1 10](#_Toc26968329)

[12.2 11](#_Toc26968330)

# Definition of terms/acronyms

| *CONSORT* | *Consolidated standards of reporting trials* |
| --- | --- |
| *MMI* | *Multimedia information resources* |
| *PIS* | *Participant information sheet* |
| *RCT* | *Randomised controlled trial* |
| *SAP* | *Statistical analysis plan* |
| *SWAT* | *Study within a trial* |

# Design

## 2.1 TRECA

The TRECA study is divided into two phases: Phase 1 (development); and Phase 2 (evaluation).

This SAP relates to Phase 2. In this evaluation phase, the MMI will be tested in a series of embedded trials hosted within healthcare trials (SWATs). The MMI will be tailored slightly for each host trial by adding a small amount of content to the MMI specific to that host trial. The MMI will be tested for impact on cognitions (i.e. decisions about trial participation taken by children and adolescents and/or parent/carer) and behaviours (rates of recruitment to, and retention in, the host trials).

This evaluation phase will use an embedded randomised controlled trial (RCT) design, with potential participants in the host healthcare trials receiving one of three versions of the recruitment information: the standard, written trial participant information sheet (PIS) alone; the standard PIS in addition to the MMI; or the MMI only. These will form the three arms of the study. The recruitment, retention and decision making data will be returned to the TRECA team from each of the six host trials, where the data from each trial will be individually analysed and then ultimately combined in meta-analyses by a statistician at the University of York.

Full details of the background and design of the TRECA study are presented in the protocol (version 2.15). Any changes between the protocol and the analysis plan are detailed.

## 2.2 Host Trials

Host trials may use individual or cluster randomisation as deemed practical and appropriate. Although the trials contains three arms, the two arms of PIS only and MMI only are the main area of scientific interest. We added the third (combined) arm as an option in case host trials or the relevant REC was concerned about ‘MMI only’ being used in recruitment.

Further information is given about each of the individual trials below, and a summary of the characteristics of each trial are given in Table 1 and Table 2. As this study looks at all those approached to enter each trial, the number of participants included in TRECA is expected to be higher than the expected sample size, as seen in Table 1.

**BALANCE**: A trial recruiting children with “Lazy eye" (amblyopia) from 3 sites (2 clinics at Moorfields Eye Hospital in London and 1 clinic in Bedfordshire). In this pilot trial, children will receive, at random, either a Nintendo 3DSXL console with movies, or standard patching/blurring eye-drop treatment.

**BAMP**: A single-centre clinical trial assessing the effectiveness of manipulate surgical treatment to bring the upper jaw forwards in 11-14 years old children. BAMP also contains a hypothetical sub study, where participants will be given information about the trial and they will then be asked if they would hypothetically enter the trial. Due to the fact that this is a hypothetical study and hence different in nature to the other host trials, the results from the sub-study will not be included in the meta-analysis. TRECA materials will be used in both recruitment to the main study and the additional hypothetical sub- study.

**CHAMP UK**: A multicentre trial placebo controlled randomised trial which investigates low-dose atropine eye drops to reduce progression of myopia in children.

**FORCE**: A multi-centre prospective randomised trial of a soft bandage and immediate discharge versus current treatment with rigid immobilisation for acute torus fractures of the distal radius in children. For the FORCE trial, cluster randomisation (by recruitment site) is used for the TRECA allocations. We will use only two arms of TRECA (PIS only versus MMI only) to enable the power to be increased and the impact of the MMIs to be more robustly evaluated.

**THERMIC-3**: A multi-centre trial which considers intermittent antegrade warm blood versus cold blood cardioplegia in children undergoing open heart surgery.

**UKALL**: The UKALL trial is a multicentre, phase III, randomised controlled trial, with two phases of randomisations. The aims are to define whether further refinement of minimal residual disease (MRD) based risk stratification and treatment regimen improves survival whilst reducing overall burden of therapy in children and young adults suffering from acute lymphoblastic leukaemia or lymphoblastic lymphoma.

Table 1 is given below outlining the main characteristics of all the host trials. Table 2 gives the details of the hypothetical study in BAMP. The type of questionnaire are as follows:

- Y = younger patient version
- O = older patient version
- P/F = patient family version

Table 1 – Host trial summary

| Trial | Target Sample Size | Expected TRECA sample size | Type of randomisation in trial | Type of randomisation in TRECA | TRECA arms being used | Decision making questionnaires used |
| --- | --- | --- | --- | --- | --- | --- |
| BALANCE | 66 | 100 | Individual | Individual | PIS, MMI or both | Y, P/F |
| BAMP | 60 | 10 | Individual | Individual | PIS, MMI or both | O, P/F |
| CHAMP-UK | 289 | 413 | Individual | Individual | PIS, MMI or both | Y, P/F |
| FORCE | 696 | 1071 | Individual | Cluster | PIS or MMI | P/F |
| THERMIC-3 | 94 | 118 | Individual | Individual | PIS, MMI or both | Y, O, P/F |
| UKALL | 40 | 50 | Individual | Individual | PIS, MMI or both | Y, O, P/F |

Table 2 – Characteristics of hypothetical BAMP sub-study

| Trial | Target Sample Size | Expected TRECA sample size | Type of randomisation in TRECA | TRECA arms being used | Decision making questionnaires used |
| --- | --- | --- | --- | --- | --- |
| BAMP sub-study | 148 | 148 | Individual | PIS or MMI | O |

# Trial Objectives

The immediate aims of TRECA are to evaluate the potential for MMIs to improve the quality of decision making about participation in healthcare trials involving children and adolescents, and to assess the impact on trial recruitment and retention.

The long-term aim of the project is to increase the available clinical evidence base for the treatment of children and adolescents, including those with long-term health conditions.

The aim of this phase of TRECA is to evaluate the MMI in a series of SWATs, and test their effects on recruitment and retention rates, and decision-making, by comparing the effects of providing standard written participant information with provision of the MMIs either in addition to the standard written participant information or the provision of the MMIs alone.

Hence, the two pairwise comparisons of the three TRECA arms of interest are whether the MMI provision could replace (MMI only vs PIS only) or supplement (MMI & PIS vs PIS only) the standard written participant information. The results of the individual trials will be combined statistically in a meta-analysis, as described in Section 8.

# Sample Size

## 4.1 Overall

The sample size for each SWAT will ultimately be determined, and constrained, by the number of people approached to take part in the host trial.

An example sample size calculation based on the expected baseline recruitment rate of the host trials (that is, their recruitment rate without the intervention), is provided. A baseline recruitment rate of 20% to 80% is assumed, to account for the known variation in trial recruitment rates. Given this uncertainty, we estimated the sample size based on the relative effect of the MMI alone (when compared to PIS alone). Further, we assumed 80% power at standard 5% Type I error (α rate) to detect the specified effect and we have characterised the effect size as an odds ratio, which is more robust for sample size calculation.

Assuming the typical recruitment rate is 20%, an odds ratio of 1.2 would mean an increase in recruitment rate using the MMI to 23.1%. To detect this in a single randomised controlled trial (with 1:1 randomisation between MMI and printed material arms), a sample size of 701 would be needed. If the typical recruitment rate is 80%, an odds ratio of 1.2 corresponds to an increase in recruitment rate to 82.8% and would require a sample size of 783.

Results from each embedded trial will ultimately be combined in a meta-analysis. Given that there will be different trials with variation in interventions, participants and baseline recruitment rates, it is plausible that the effectiveness of the MMIs at improving recruitment will vary; i.e. there will be heterogeneity in the observed odds ratios across trials. Adjusting for this is approximate (particularly as the heterogeneity is currently unknown), however, as a rough rule of thumb if the I2 statistic in the meta-analysis is 50% the sample size will double (1).

Given the three arm randomisation (with two of the arms being compared for the primary analysis), an additional 50% of people will need to be approached (i.e. 783 x 1.5 = 1,175). Furthermore, the adjustment for heterogeneity in effects of the MMI intervention across the six trials (estimated I-square value = 50%) means that the sample size should be doubled (i.e. 1,175 x 2 = 2,350).

Therefore, the six embedded trials in TRECA should (on average) each be approaching 392 people, assuming a baseline trial recruitment rate between 20% and 80% of those approached. We will make pragmatic decisions about inclusion of each host trial into TRECA, in part to attain variation among the included trials in aspects of the intervention and setting, and in part to ensure that we recruit a full complement of embedded trials to TRECA. Further, trials involving children and adolescents are often relatively small: our random sample survey of 100 trials involving children and adolescents with long-term conditions (2) found that only two-thirds had a target sample size of >216. Given the other entry criteria applied to host trials, one or more of the host trials recruited to TRECA may have a smaller target sample size than 216. Consequently the meta-analysis of the 6 embedded trials may be required to provide a robust evaluation of the MMIs’ effectiveness.

## 4.2 BAMP hypothetical sub-study

We will seek to recruit 148 participants to this sub-study (74 in PIS group and 74 in MMI group). This is to allow for 20% of those randomised not being able to complete the questionnaires (e.g. due to time available or not completing all questionnaires). The questionnaires have 9 Likert scale questions with each of these questions having a score option of 0-4, so the total possible score range is 0-36 (for example 0 means very hard and 4 means very easy; Please see appendix). A difference between groups (MMI versus PIS) of 4.5 (reflecting a mean of 0.5 point different on each of the 9 questions with a Likert scale) would be meaningful. Standard deviation (SD) on the scale of pooled scores is 6.75 (estimated that 95% scores would fall between 4.5 and 31.5 is 27, dividing 27 by 4 for approximate SD = 6.75). Power is 90% and significance level = 0.01. Further information about the questionnaires is given in Section 6.2.3.

# Randomisation

Allocation to groups will be achieved by random number generator or another randomisation method that suits the practicalities of the host trial. Participants will be randomised 1:1 to either PIS or MMI (two arms) in the BAMP sub-study. In FORCE clusters (hospitals) are randomised to either PIS or MMI. Otherwise, participants will be randomised 1:1:1 to one of PIS, MMI or both.

Masking of the allocation at outcome measurement is not possible but also irrelevant: the patient cannot be masked to the information format s/he will receive but, as s/he will be unaware of the embedded information trial, a lack of masking will not affect his/her responses on the self-completion measures, or have any biasing effect on their decisions on trial participation or continuation.

# Outcomes

## 6.1 Primary outcome (Recruitment - MMI only vs PIS only)

The primary outcome is recruitment to each host trial between the MMI only arm and the PIS only arm. For recruitment we will calculate the proportion of patients who agree to participate from the total approached, for each arm of the embedded trial. We will assume that patient eligibility for host trial participation will have been assessed before an approach has been made.

Data on recruitment to the host trial will be recorded automatically within the host trial dataset.

## 6.2 Secondary outcomes

### 6.2.1 Recruitment (MMI & PIS vs PIS only)

The secondary recruitment outcome is recruitment to each host trial between the PIS & MMI arm and the PIS only arm. For recruitment, we will calculate the proportion of patients who agree to participate from the total approached, for each arm of the embedded trial. We will assume that patient eligibility for host trial participation will have been assessed before an approach has been made.

### 6.2.2 Retention

For the retention outcome, we will obtain data on the number and timing of drop outs from each host trial. A single time point specified in the final column in Table 3 will be used in the analyses. Data on trial retention will also be recorded automatically within the host trial dataset.

Table 3 – Summary of retention time points

| Trial | Follow – up time points | Time point for assessing retention |
| --- | --- | --- |
| BALANCE | 16 weeks follow up time point | 16 weeks |
| BAMP | 18 months and 3 years | 18 months |
| CHAMP UK | 6, 12, 18, 24 months (primary outcomes); 5 years post randomisation | 6 months |
| FORCE | 1 day, 3 days, 7 days, 21 days, 6 weeks (eCRF via text msg or telephone) | 6 weeks |
| THERMIC-3 | 3 months | 3 months |
| UKALL | After each course of intensive therapy and 3 monthly whilst on therapy | 3 months |

### 6.2.3 Quality of Decision Making

We will also measure the quality of decision making by potential host trial participants. Children and adolescents will be asked to complete a brief decisional scale, adapted from one used within the REFORM trial (unpublished data; P Knapp, P Bower, J Graffy, J Rick, S Cockayne) and drawing conceptually on the SURE (3, 4) and DelibeRATE scales (5). When a parent/carer has been involved in the participation decision, we will also ask them to complete the scale. The scale has been adapted to facilitate completion by young children. We will aim to obtain decision quality scores both from individuals who decide to participate in the host trial and those who decline.

In patients who decide to take part, the children and adolescents and/or parent/carer will be asked to complete the decisional scale once the host trial participation documentation has been completed. In patients who decline participation, they will be asked to complete this measure in the clinic or will have them posted at home or emailed, as appropriate.

The adolescent and parent/family versions of the questionnaire contain the same number of questions, with slight changes in the phrasing of questions. The decisional scale contains 9 Likert questions (with 5 possible answer options) and there are a further 3 questions containing space for free text which give participants the opportunity to give further opinion on their experience. There is also a question detailing who filled in the questionnaire (‘patient’ or ‘parent/carer and patient together’). The younger patient version of the questionnaire is of a similar format. The decisional scale contains 3 Likert questions (each with 5 possible answer options) and the further 3 open ended questions (Please see appendix).

The decisional scales will be scored for both the 3 and 9 question version. Answers to each Likert question will be allocated a value of 0-4. The values for each question will be summed to create an overall score, out of 12 and 36, for the two versions of the scale. Up to three missing responses will be allowed on the 9 question scale. One missing value will be allowed on the 3 question version of the scale. A total score will be calculated by replacing the missing values with the mean score from the completed responses given by the participant. Any scores with more than 3 (adolescent and parent/family version) or more than 1 (younger patient version) will not be scored.

## 6.3 Other important information

In order to assess any potential moderating influences of other variables on the effectiveness of the MMIs, we will aim to obtain data within each host trial of children and adolescents’ age, gender, and deprivation score, according to allocation in the embedded trial and to host trial participation decision.

# Analysis

A CONSORT diagram displaying the flow of participants through each trial will be reported.

All analyses will be conducted in STATA v16 (StataCorp, 4905 Lakeway Drive, College Station, Texas 77845 USA), or later (to be confirmed in final report), following the principles of intention-to-treat with participant’s outcomes analysed according to their original, randomised group. The following sections relate to the separate analyses of the host trials. Details of the meta-analyses are given in Section 8. Analysis of the primary outcome will be checked by a second statistician after they are conducted. The data relating to the main BAMP study and the BAMP sub-study will be analysed separately. Appropriate model diagnostics will be assessed when models are fitted, including checking normality (for continuous outcomes), checking normality of random effects and checking the homoscedasticity of residuals.

As all the analyses include the PIS only arm, it is important to consider the effect of this on the Type 1 error rate. Here, we will not adjust for multiplicity as we only have a single primary outcome comparison (rather than a co-primary outcome or a primary outcome that is a combination of the multiple comparisons).

## 7.1 Baseline data

All participant baseline data will be summarised descriptively by TRECA trial arm. No formal statistical comparisons will be undertaken. Continuous measures will be reported as means and standard deviations (normality will be checked and if non-normal medians and interquartile ranges will be reported) and categorical data will be reported as counts and percentages. Baseline data for some of the host trials will vary due to different trial data collection, all data that is collected will be reported. The baseline data that will be collected for each trial is given in Table 5 in the Appendix. Baseline data will only be available for participants who are randomised into each trial.

## 7.2 Primary analysis (Recruitment - MMI only vs PIS only)

The proportion of eligible patients entering the trial, which is defined as the number randomised over the number of eligible participants approached will be reported by SWAT trial arm. Recruitment rates will be compared using logistic regression, with TRECA allocation included as covariate in the model. Clustering will be accounted for in the analysis of FORCE, by including the cluster variable as a random effect. The primary comparison is between the MMI only arm and the PIS only arm, hence this pairwise comparison will be extracted from the model. The results from the regression will be presented as odds ratios, with associated 95% confidence intervals and p-values.

## 7.3 Secondary analyses

### 7.3.1 Recruitment (MMI & PIS vs PIS only)

The secondary outcome is looking at the effect of the addition of MMI to PIS. This pairwise comparison will be extracted from the same model as was used for the primary analysis. This outcome is not applicable for FORCE as they only used the MMI and PIS arms.

### 7.3.2 Retention

The time point for assessing in each trial is given in Table 3. The proportion of the participants who are retained at the specified time point will be reported. This is defined as the number of participants who have reached that time point, over the number of participants randomised into the trial.

The retention rate will be compared using logistic regression, for each host trial, with host trial allocation and TRECA allocation included as covariates. Where a host trial has used stratification variables (see Table 4) in the randomisation, these will be included as covariates in the model. Clustering will be accounted for in the analysis of FORCE, by including the cluster variable as a random effect. Similarly to the recruitment analyses, two pairwise comparisons will be used: MMI only vs PIS only and MMI & PIS vs PIS only. The results will be presented as odds ratios, with associated 95% confidence intervals, and p-values.

Table 4 – Summary of stratification factors

| Trial | Stratification factors |
| --- | --- |
| BALANCE | Type of amblyopia & centre by stratification |
| BAMP | Gender by stratification |
| CHAMP UK | Centre, ethnicity, severity of myopia. The unit of randomisation will be the participant (not the eye) using a minimisation algorithm |
| FORCE | Age & centre by stratification |
| THERMIC-3 | Risk Adjustment for Congenital Heart Surgery (RACH scores) by stratification |
| UKALL | Cytogenetic risk and Minimal residual disease (MRD) level, National Cancer Institute (NCI) Risk, early morphologic response by stratification |

### 7.3.3 Quality of Decision Making

The responses to each question (including the amount of missing responses) and the calculated total scores of the decisional questionnaire will be summarised descriptively overall and broken down by host trial, TRECA allocation and type of questionnaire (younger patient, adolescent or parent/family). A bar chart showing the total scores for each of the three TRECA arms will be included.

As a patient and their parent/carer may both fill in a questionnaire, all the data from the all three questionnaires will not be combined, as all the scores would not be independent. Hence, scores for patients (younger or older) and parents/family questionnaires will be analysed separately using a linear regression, with TRECA allocation and host trial status (whether the participant went on to enter the trial) included as a covariates. Clustering will be accounted for by using the cluster variable as a random effect. Mean difference will be presented with 95% confidence intervals.

If both the younger and older patient questionnaires are used in a host trial the questionnaire data from the younger and older patient questionnaires will be combined. The scores for these will be standardised within the trial by subtracting the sample mean score from the observed score and dividing the result by the sample standard deviation, where the ‘sample’ refers to the subset of participants that have used that questionnaire. Then the standard scores for all participants will be analysed as described above.

To assess the robustness of the method used to replace the missing values, a sensitivity analysis will be conducted, where the analysis will be repeated using only the questionnaires in which all 9 questions were answered.

Both the above analyses (in this section) will be repeated for only the participants who went onto to enter a trial.

# Meta-analyses

Results from each embedded trial will ultimately be combined in meta-analyses. The BAMP sub-study will not be included in these meta-analyses due to its hypothetical nature. The meta-analyses will be displayed graphically using forest plots, which display the effect size estimates and confidence intervals for each trial in addition to the overall result.

## 8.1 Recruitment

Two random effects meta-analyses will be conducted using the odds ratio for each of the host trials which were calculated in the recruitment primary analysis (PIS only vs MMI only) and the recruitment secondary analysis (MMI & PIS vs PIS only). FORCE cannot be included in the second meta-analysis as only two arms (PIS only and MMI only) were used in the trial. The I2 statistic will be used to assess the heterogeneity between the trials. A sensitivity analysis for each meta-analysis will be carried out, in which a fixed-effects model will be used.

## 8.2 Retention

Two random effects meta-analyses will be conducted using the odds ratio for each of the host trials which were calculated in the retention secondary analyses. The I^2^ statistic will be used to assess the heterogeneity between the trials. A sensitivity analysis for each meta-analysis will be carried out, in which a fixed-effects model will be used.

## 8.3 Quality of decision making

The decision making data from all six trials (including the BAMP main trial, but not the BAMP sub-study) will also be combined in four meta-analyses, which will mirror the four analyses specified in section 7.3.2. Mean difference scores from each of the trials will be combined in a random effects meta-analysis. A sensitivity analysis for each meta-analysis will be carried out, in which a fixed-effects model will be used.

# SAP amendment log

All the changes that are made to the Statistical Analysis Plan following initial sign-off in the box below.

| **Amendment/addition to SAP and reason for change** | **New version number, name and date** |
| --- | --- |
| SAP completed and signed-off | *V1.4, 21/01/2020* |
|  |  |
|  |  |
|  |  |

# Signatures of approval

Sign-off for the final approved version of the Statistical Analysis Plan by the principle investigator and trial statistician(s) (can also include Trial Manager/Co-ordinator)

| **Name** | **Trial Role** | **Signature** | **Date** |
| --- | --- | --- | --- |
| Jenny Roche | Statistician | 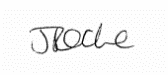 | 21/01/2020 |
| Peter Knapp | Principal Investigator |  | 31^st^ January 2020 |
|  |  |  |  |

# References

1. Wetterslev J, Thorlund K, Brok J, Gluud C. Estimating required information size by quantifying diversity in random-effects model meta-analyses. BMC medical research methodology. 2009;9(1):86.

2. ISRCTN. <http://www.controlled-trials.com/> 14 May 2014

3. Ferron Parayre A, Labrecque M, Rousseau M, Turcotte S, Legare F. Validation of SURE, a four-item clinical checklist for detecting decisional conflict in patients. Medical Decision Making. 2014;34(1):54-62.

4. Légaré F, Kearing S, Clay K, Gagnon S, D’Amours D, Rousseau M, et al. Are you SURE?: Assessing patient decisional conflict with a 4-item screening test. Canadian family physician. 2010;56(8):e308-e14.

5. Gillies K, Elwyn G, Cook J. Making a decision about trial participation: the feasibility of measuring deliberation during the informed consent process for clinical trials. Trials. 2014;15(1):307.

# Appendix

## 12.1

Table 5 contains details of the baseline data that will be received from each of the host trials.

Table 5 – Baseline data collected

| Trial | Baseline data expected |
| --- | --- |
| BALANCE | Site, main trial allocation, age, gender, ethnicity, parent/family who provides consent gender, parent/family who provides consent age, whether English is first language of parent/family who provides consent, deprivation index for home address |
| BAMP | Main trial allocation, age, deprivation index for home address |
| CHAMP UK | Site, main trial allocation, age, gender, ethnicity, parent/family who provides consent gender, deprivation index for home address |
| FORCE | Site, main trial allocation, age, gender, ethnicity, whether English is first language, deprivation index for home address |
| THERMIC-3 | Main trial allocation, age, gender, deprivation index for home address |
| UKALL | Main trial allocation, age, gender, ethnicity, deprivation index for home address |

## 12.2

This appendix contains two examples of the decision making questionnaires that have been used in the UKALL trial. The first is for 5-11 year olds (Younger patient version) and the second is for 12-18 year olds (Older patient version).

**Add hospital header/logos here**

**Decision-making Questionnaire**

**(Younger patient version)**

*For office use only*

Trial site:
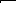


Participant trial ID number

(if applicable):

**/**

**/**

Date questionnaire sent:

Years

Participant’s Age:

--------------------------------------------------------------------------

We would like to know what you think about the information you were given about the UKALL 2011 trial and how much it helped your decision about taking part in the trial. We would still like your views on the information, whether you decided to take part in the trial or not.

Firstly, did you read or view **any** information about UKALL 2011 trial before making your decision about whether or not to take part?

Yes, I read some information on paper

Yes, I viewed some information on a computer or phone

Yes, I read some information on paper **and**

on a computer or phone

No, I did not read or view any of the information

**If you answered ‘yes’, please answer a few questions for us on the next couple of pages.**

**------------------------------------------------------------------------------------------------------------------------------**

**1) The information I saw about the UKALL 2011 trial was easy to understand.**

Very hard Hard OK Easy Very easy


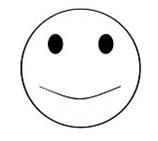

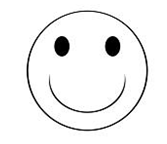

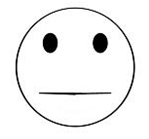

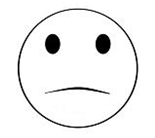


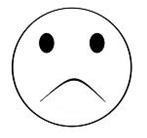


**2) After seeing the information about the UKALL 2011 trial I knew what taking part would be like.**

Not at all Not really Not sure Yes, mostly Yes, completely


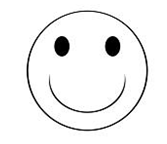

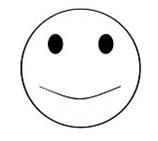

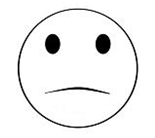

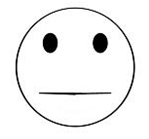

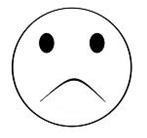


**3) The information I saw about the UKALL 2011 trial helped me decide if I wanted to take part.**

No Not really Not sure A bit Yes


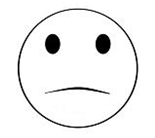

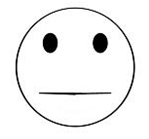

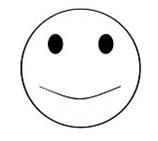

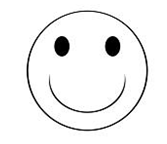

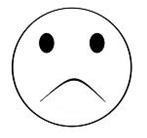


**4) Was there anything you wanted to know about UKALL 2011 trial but which wasn’t included in the information you saw?** Please tell us about this.

**5) Was there anything in the information you saw about UKALL 2011 trial that was explained well?** Please tell us about this.

**6) Was there anything in the information you saw about UKALL 2011 trial that you found interesting?** Please tell us about this.

**Thank you for taking the time to complete this questionnaire.**

**Please return it to your research nurse on the day of your visit.**

**Add hospital header/logos here**

**Decision-making Questionnaire**

**(Older patient version)**

*For office use only*

Trial site:
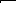


Participant trial ID number

(if applicable):

**/**

**/**

Date questionnaire sent:

Years

Participant’s Age:

--------------------------------------------------------------------------

We would like to know your views on the information you were given about the UKALL 2011 trial and how much it helped your decision about taking part in the trial. We would still like your views on the information, whether you decided to take part in the trial or not.

Firstly, did you read or view **any** information about the UKALL 2011 trial before making your decision about whether or not to take part?

Yes, I read some information on paper Go to Section 1

Yes, I viewed some information on a computer or phone Go to Section 1

Yes, I read some information both on paper **and** Go to Section 1

on a computer or phone

No, I did not read or view any of the information Go to Section 2

**------------------------------------------------------------------------------------------------------------------------------Section 1:**

Listed below are 12 statements about the information that you were given for the UKALL 2011 trial. For each statement please **put a circle** round the option that best matches your view. In other words, show how much you agree or disagree with the statement.

**1) The information I saw about the UKALL 2011 trial was easy to understand.**

Very hard Hard OK Easy Very easy

**2) After seeing the information about the UKALL 2011 trial I knew what taking part would be like.**

Not at all Not really Not sure Yes, mostly Yes, completely

**3) The information helped me understand how my treatment or care might change if I took part in the UKALL 2011 *trial*.**

Not at all Not really Not sure Yes, mostly Yes, completely

**4) The possible benefits of taking part in the *UKALL 2011 trial* were made clear in the information.**

Not at all Not really Not sure Yes, mostly Yes, completely

**5) The possible disadvantages of taking part in the UKALL 2011 trial were made clear in the information.**

Not at all Not really Not sure Yes, mostly Yes, completely

**6) The information about the UKALL 2011 trial helped me discuss the trial with the person who asked me to take part (usually a doctor, nurse or researcher).**

Not at all Not really Not sure Yes, mostly Yes, completely

**7) The information about the UKALL 2011 trial helped me discuss taking part with my parent(s) or family.**

Not at all Not really Not sure Yes, mostly Yes, completely

**8) I am confident that I have made the right decision about whether or not to take part in the UKALL 2011 trial.**

Not at all Not really Not sure Yes, mostly Yes, completely

**9) In all, the information about the UKALL 2011 trial helped me make my decision about whether or not to take part.**

Not at all Not really Not sure Yes, mostly Yes, completely

**10) Was there anything you wanted to know about UKALL 2011 trial but which wasn’t included in the information you saw? Yes / No (please circle).**

**If yes, please write them here:**

**11) Can you tell us which aspect(s) about UKALL 2011 trial was explained well in the information you saw? Please write them here:**

**Section 2:**

**If you have any other comments about the information you were given about the UKALL 2011 trial, please write them here:**

**Finally, please let us know who completed this questionnaire by ticking one box below.**

Patient: Parent/carer and patient together:

Other (please specify):…………………..

**Thank you for taking the time to complete this questionnaire.**

**Please return it to your research nurse on the day of your visit.**
