# Supplementary material for: Providing multimedia information to children and young people increases recruitment to trials: pre-planned meta-analysis of SWATs
Source: BMC Med. 2023 Jul 4;21:244. doi: 10.1186/s12916-023-02936-1 (PMC10320935; doi:10.1186/s12916-023-02936-1)
Supplement: Supplementary file 2 — Additional file 2. Baseline characteristics for participants who consented to the FORCE, CHAMP-UK and Thermic-3 trials. [file 12916_2023_2936_MOESM2_ESM.docx]

**Supplementary Material (2)**

**Baseline characteristics for participants who consented to the FORCE, CHAMP-UK and Thermic-3 trials**

Baseline characteristics for participants who consented to the FORCE trial

|  | **PIS-only (n=484)** | **MMI-only (n=475)** | **Overall**  **(n=959)** |
| --- | --- | --- | --- |
| **Age (child)**  n (n missing)  Mean (SD) | 484 (0)  9.3 (2.8) | 475 (0)  9.0 (3.0) | 959 (0)  9.1 (2.9) |
| **Gender (child), n (%)**  Male  Female | 302 (62.4)  182 (37.6) | 280 (59.0)  195 (41.1) | 582 (60.7)  377 (39.3) |
| **Ethnicity, n (%)**  Asian/Asian British  Black/African/Caribbean/Black British  White  Mixed/multiple ethnic groups  Other ethnic group  Not stated | 66 (13.6)  28 (5.8)  361 (74.6)  10 (2.1)  15 (3.1)  4 (0.8) | 31 (6.5)  20 (4.2)  408 (85.9)  9 (1.9)  6 (1.3)  1 (0.2) | 97 (10.1)  48 (5.0)  769 (80.2)  19 (2.0)  21 (2.2)  5 (0.5) |
| **English as first language, n (%)**  Yes  No  Missing | 439 (90.7)  43 (8.9)  2 (0.4) | 452 (95.2)  23 (4.8)  0 (0.0) | 891 (92.9)  66 (6.9)  2 (0.2) |
| **Deprivation index for home address decile**^a^  n (missing)  Median (p25, p75) | 484 (0)  5 (2, 7) | 474 (1)  4 (2, 7) | 958 (1)  4 (2, 7) |

Baseline characteristics for participants who consented to the CHAMP-UK trial

|  | **PIS**  **(n=29)** | **MMI**  **(n=38)** | **MMI & PIS (n=29)** | **Overall**  **(n=96)** |
| --- | --- | --- | --- | --- |
| **Age (child)**  n (n missing)  Mean (SD) | 29 (0)  8.8 (1.5) | 37 (1)  9.2 (1.8) | 29 (0)  9.4 (1.5) | 95 (1)  9.2 (1.6) |
| **Gender (child), n(%)**  Male  Female  Missing | 15 (51.7)  14 (48.3)  0 (0.0) | 18 (47.4)  19 (50.0)  1 (2.6) | 18 (62.1)  11 (37.9)  0 (0.0) | 51 (53.1)  44 (45.8)  1 (1.0) |
| **Ethnicity, n (%)**  Asian/Asian British  Black/African/Caribbean/Black British  White  Mixed/multiple ethnic groups  Other ethnic group  Not stated/Missing | 6 (20.7)  2 (6.9)  14 (48.3)  1 (3.5)  5 (17.2)  1 (3.5) | 4 (10.4)  1 (2.6)  25 (65.8)  6 (15.8)  0 (0.0)  2 (5.2) | 1 (3.5)  1 (3.5)  23 (79.3)  0 (0.0)  4 (13.8)  0 (0.0) | 11 (11.5)  4 (4.2)  62 (64.6)  7 (7.3)  9 (9.4)  3 (3.1) |
| **Gender (parent), n (%)**  Male  Female  Missing | 12 (41.4)  16 (55.2)  0 (0.0) | 8 (21.1)  29 (76.3)  1 (2.6) | 12 (41.4)  17 (58.6)  0 (0.0) | 32 (33.3)  62 (64.6)  1 (1.0) |

Participant baseline characteristics for those randomised to the Thermic-3 trial

|  | **PIS-only**  **(n = 47)** | **MMI-only**  **(n = 49)** | **MMI & PIS (n = 51)** | **Overall**  **(n = 147)** |
| --- | --- | --- | --- | --- |
| **Age (child)**  n (missing)  Median (p25, p75) | 47 (0)  0.75 (0.25, 4.33) | 49 (0)  0.5 (0.33, 3.33) | 51 (0)  0.91 (0.33, 3.75) | 147 (0)  0.67 (0.33, 3.75) |
| **Gender (child), n (%)**  Male  Female | 30 (64)  17 (36) | 28 (57)  21 (43) | 33 (65)  18 (35) | 91 (62)  56 (38) |
| **Deprivation index for home address decile**^a^  n (missing)  Median (p25, p75) | 47 (0)  4 (1, 8) | 49 (0)  5 (2, 6) | 51 (0)  2 (1, 6) | 147 (0)  4 (1, 7) |
