# Supplementary material for: Providing multimedia information to children and young people increases recruitment to trials: pre-planned meta-analysis of SWATs
Source: BMC Med. 2023 Jul 4;21:244. doi: 10.1186/s12916-023-02936-1 (PMC10320935; doi:10.1186/s12916-023-02936-1)
Supplement: Supplementary file 3 — Additional file 3. Results of the CHAMP-UK SWAT. [file 12916_2023_2936_MOESM3_ESM.docx]

**Supplementary Material (3)**

**Results of the CHAMP-UK-UK SWAT**

Participants were recruited at four sites for TRECA within the CHAMP-UK-UK SWAT. In total, 208 participants were randomised, but 7 (3.4%) were subsequently found to be ineligible for CHAMP-UK-UK, and so the analysis comprises 201 participants: 65 PIS, 68 MMI and 68 MMI & PIS. 96 (47.8%) of the 201 participants were consented to the CHAMP-UK-UK trial.

In the MMI arm, 38 out of 68 participants consented to CHAMP-UK-UK (55.9%), compared to 28 out of 65 (43.1%) in the PIS arm. A logistic regression model produced an OR = 1.67 (95% CI 0.84, 3.32, p=0.14), suggesting that those in the MMI arm were more likely to be recruited, but this difference was not statistically significant.

There were similar recruitment rates for the combined MMI & PIS (30/68; 44.1%) and the PIS-only arms (28/65; 43.1%). A logistic regression model produced an OR = 1.04 (95% CI 0.53, 2.07, p=0.90), suggesting a very small increase in the odds of being recruited for those receiving combined MMI & PIS, but the result was not statistically significant.

Of the 96 participants who consented to CHAMP-UK-UK through TRECA, 89 (92.7%) completed their follow-up: 26 PIS (92.9%, of 28), 34 MMI (98.5%, of 38), and 29 MMI & PIS (96.7%, of 30).

The retention analysis for PIS vs MMI was adjusted for TRECA allocation (PIS or MMI), severity of myopia (>=3D, or < 3D), and ethnicity (white or non-white), as these were used in the minimisation for the host trial randomisation. Trial site was also used in the minimisation but was omitted in this analysis because it allowed the inclusion of more participants in the model. It was not possible to adjust for host trial allocation, as this was not available at the time of analysis (CHAMP-UK-UK was a double-blind trial and had not been analysed when the SWAT data were needed). The results were an OR of 1.11 (95% CI 0.12 to 10.27, p=0.92), which suggest a small positive benefit to retention for those who received MMI compared to the PIS, however, the results were not statistically significant.

When analysing the retention rate for PIS-only compared to combined MMI & PIS, the model was only adjusted for TRECA allocation, to improve accuracy of results, by increasing the number of participants. An OR of 2.23 was found (95% CI 0.19 to 26.06, p=0.52), which suggests a benefit to retention when the MMI was used in addition to the PIS, compared to PIS alone, but again the result is not statistically significant.

Decision-Making Questionnaires were received from 81 participants: 55 who consented to the CHAMP-UK-UK trial and 16 who declined consent. The DMQ return rate was 27.4% among consenters and 15.2% among decliners. Mean DMQ scores were 2.5 points higher in the PIS arm when compared to the MMI arm, with the results statistically significant (n= 57; AMD: -2.43; 95% CI -4.61, -0.24; p=0.03). When the combined MMI & PIS arm was compared with the PIS-only arm, mean DMQ scores were 2.2 points higher in the PIS arm, and the difference is statistically significant (n=50; AMD: -2.11; 95% CI -4.23, 0.01; p=0.05).

Analysing only those participants who had fully completed the DMQ measure, the same pattern of results is evident: scores were 2.6 points higher in the PIS arm than in the MMI arm (n=56; AMD: -2.53; 95% CI -4.73, -0.32; p=0.03) and scores were 2.5 points higher in the combined MMI & PIS arm than in the PIS-only arm (n=48; AMD: -2.38; 95% CI -4.52, -0.23; p=0.03). Both differences are statistically significant.
