# Supplementary material for: Providing multimedia information to children and young people increases recruitment to trials: pre-planned meta-analysis of SWATs
Source: BMC Med. 2023 Jul 4;21:244. doi: 10.1186/s12916-023-02936-1 (PMC10320935; doi:10.1186/s12916-023-02936-1)
Supplement: Supplementary file 4 — Additional file 4. Decision-making Questionnaire. [file 12916_2023_2936_MOESM4_ESM.docx]

**Supplementary Material (4)**

**Decision-making Questionnaire**

**(Younger patient version)**

We would like to know what you think about the information you were given about the Thermic-3 trial and how much it helped your decision about taking part in the trial. We would still like your views on the information, whether you decided to take part in the trial or not.

Firstly, did you read or view **any** information about the Thermic-3 trial before making your decision about whether or not to take part?

Yes, I read some information on paper

Yes, I viewed some information on a computer or phone

Yes, I read some information on paper **and**

on a computer or phone

No, I did not read or view any of the information

**If you answered ‘yes’, please answer a few questions for us on the next couple of pages.**

**------------------------------------------------------------------------------------------------------------------------------**

*For office use only*

Trial site:
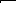


Participant’s trial ID number:

**/**

**/**

Date questionnaire sent:

Years

Participant’s Age:

**1) The information I saw about the Thermic-3 trial was easy to understand.**

Very hard Hard OK Easy Very easy


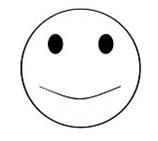

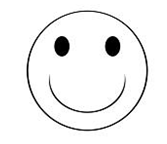

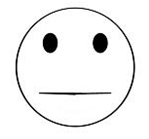

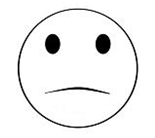


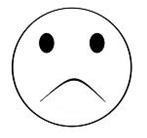


**2) After seeing the information about the Thermic-3 trial I knew what taking part would be like.**

Not at all Not really Not sure Yes, mostly Yes, completely


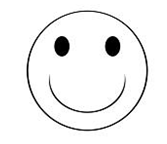

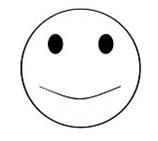

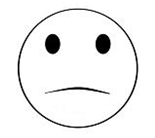

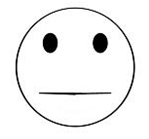

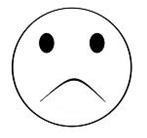


**3) The information I saw about the Thermic-3 trial helped me decide if I wanted to take part.**

No Not really Not sure A bit Yes


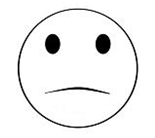

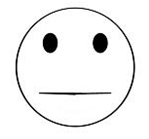

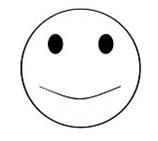

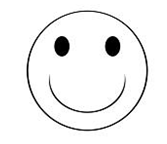

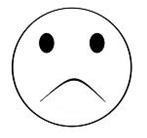


**4) Was there anything you wanted to know about the Thermic-3 trial but which wasn’t included in the information you saw?** Please tell us about this.

**5) Was there anything in the information you saw about the Thermic-3 trial that was explained well?** Please tell us about this.

**6) Was there anything in the information you saw about the Thermic-3 trial that you found interesting?** Please tell us about this.

**Thank you for taking the time to complete this questionnaire.**

**Please return it to *(insert name)* in the stamped addressed envelope provided.**

**Decision-making Questionnaire**

**(Older patient version)**

We would like to know your views on the information you were given about the Thermic-3 trial and how much it helped your decision about taking part in the trial. We would still like your views on the information, whether you decided to take part in the trial or not.

Firstly, did you read or view **any** information about the Thermic-3 trial before making your decision about whether or not to take part?

Yes, I read some information on paper Go to Section 1

Yes, I viewed some information on a computer or phone Go to Section 1

Yes, I read some information both on paper **and** Go to Section 1

on a computer or phone

No, I did not read or view any of the information Go to Section 2

**------------------------------------------------------------------------------------------------------------------------------**

*For office use only*

Trial site:
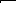


Participant’s trial ID number:

**/**

**/**

Date questionnaire sent:

Years

Participant’s Age:

**Section 1:**

Listed below are 12 statements about the information that you were given for the Thermic-3 trial*.* For each statement please **put a circle** round the option that best matches your view. In other words, show how much you agree or disagree with the statement.

**1) The information I saw about the Thermic-3 trial was easy to understand.**

Very hard Hard OK Easy Very easy

**2) After seeing the information about the Thermic-3 trial I knew what taking part would be like.**

Not at all Not really Not sure Yes, mostly Yes, completely

**3) The information helped me understand how my treatment or care might change if I took part in the Thermic-3 trial.**

Not at all Not really Not sure Yes, mostly Yes, completely

**4) The possible benefits of taking part in the Thermic-3 trial were made clear in the information.**

Not at all Not really Not sure Yes, mostly Yes, completely

**5) The possible disadvantages of taking part in the Thermic-3 trial were made clear in the information.**

Not at all Not really Not sure Yes, mostly Yes, completely

**6) The information about the Thermic-3 trial helped me discuss the trial with the person who asked me to take part (usually a doctor, nurse or researcher).**

Not at all Not really Not sure Yes, mostly Yes, completely

**7) The information about the Thermic-3 trial helped me discuss taking part with my parent(s) or family.**

Not at all Not really Not sure Yes, mostly Yes, completely

**8) I am confident that I have made the right decision about whether or not to take part in the Thermic-3 trial.**

Not at all Not really Not sure Yes, mostly Yes, completely

**9) In all, the information about the Thermic-3 trial helped me make my decision about whether or not to take part.**

Not at all Not really Not sure Yes, mostly Yes, completely

**10) Was there anything you wanted to know about the Thermic-3 trial but which wasn’t included in the information you saw? Yes / No (please circle).**

**If yes, please write them here:**

**11) Can you tell us which aspect(s) about the Thermic-3 trial was explained well in the information you saw? Please write them here:**

**Section 2:**

**If you have any other comments about the information you were given about the *(insert trial name),* please write them here:**

**Finally, please let us know who completed this questionnaire by ticking one box below.**

Patient: Parent/carer and patient together:

Other (please specify):…………………..

**Thank you for taking the time to complete this questionnaire.**

**Please return it to *(insert name)* in the stamped addressed envelope provided.**

**Decision-making Questionnaire**

**(Parent or family version)**

We would like to know your views on the information you were given about the Thermic-3 trial and how much it helped your decision about taking part in the trial. We would still like your views on the information, whether you decided to take part in the trial or not.

Firstly, did you read or view **any** information about the Thermic-3 trial before making your decision about whether or not to take part?

Yes, I read some information on paper Go to Section 1

Yes, I viewed some information on a computer or phone Go to Section 1

Yes, I read some information both on paper **and** Go to Section 1

on a computer or phone

No, I did not read or view any of the information Go to Section 2

**------------------------------------------------------------------------------------------------------------------------------**

*For office use only*

Trial site:
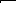


Participant’s trial ID number:

**/**

**/**

Date questionnaire sent:

Years

Participant’s Age:

**Section 1:**

Listed below are 12 statements about the information that you were given for the Thermic-3 trial*.* For each statement please **put a circle** round the option that best matches your view. In other words, show how much you agree or disagree with the statement.

**1) The information I saw about the Thermic-3 trial** **was easy to understand.**

Very hard Hard OK Easy Very easy

**2) The information helped me understand what it would be like for my son or daughter to take part in the Thermic-3 trial.**

Not at all Not really Not sure Yes, mostly Yes, completely

**3) The information helped me understand how my son’s or daughter’s treatment or care might change if s/he took part in the Thermic-3 trial.**

Not at all Not really Not sure Yes, mostly Yes, completely

**4) The possible benefits of taking part in the Thermic-3 trial were made clear in the information.**

Not at all Not really Not sure Yes, mostly Yes, completely

**5) The possible disadvantages of taking part in the Thermic-3 trial were made clear in the information.**

Not at all Not really Not sure Yes, mostly Yes, completely

**6) The information about the Thermic-3 trial helped me discuss the trial with the person who asked my son or daughter to take part (usually a doctor, nurse or researcher).**

Not at all Not really Not sure Yes, mostly Yes, completely

**7) The information about the Thermic-3 trial helped me discuss taking part with my son or daughter.**

Not at all Not really Not sure Yes, mostly Yes, completely

**8) I am confident that I have made the right decision about whether or not my son or daughter should take part in the Thermic-3 trial.**

Not at all Not really Not sure Yes, mostly Yes, completely

**9) In all, the information about the Thermic-3 trial helped me make my decision about whether or not my son or daughter should take part.**

Not at all Not really Not sure Yes, mostly Yes, completely

**10) Was there anything you wanted to know about the Thermic-3 trial but which wasn’t included in the information you saw? Yes / No (please circle).**

If yes, please write them here:

**11) Can you tell us which aspect(s) about the Thermic-3 trial was explained well in the information you saw?** Please write them here:

**Section 2:**

**If you have any other comments about the information you were given about the Thermic-3 trial*,* please write them here:**

**Finally, please let us know who completed this questionnaire by ticking one box below.**

Patient: Parent/carer and patient together:

Other (please specify):…………………..

**Thank you for taking the time to complete this questionnaire.**

**Please return it to *(insert name)* in the stamped addressed envelope provided.**
